# Supplementary material for: Feasibility of a patient-oriented navigation programme for patients with lung cancer or stroke in Germany: Protocol of the CoreNAVI study
Source: PLoS One. 2023 Jun 29;18(6):e0287638. doi: 10.1371/journal.pone.0287638 (PMC10309639; doi:10.1371/journal.pone.0287638)
Supplement: S1 File — (PDF) [file pone.0287638.s002.pdf]

Translation of ethics protocol main text (commented version):

Application for consultation of the ethics committee the investigation of a medical-scientific project that does not involve the clinical testing of a drug or medical device.

Study title	CoreNAVI- Investigating the feasibility of a patient-centered navigation model in stroke and lung cancer
1. Decisions of other ethics committees in the same matter	No
2. Subject of the study and its aims, specification of the hypotheses, separated into main and secondary hypotheses and the clinical parameters (primary and secondary endpoints), which will be used to test the hypotheses	Primary Aim:<br>In a mixed-methods study in the design of two two-arm randomized controlled trials with parallel cohort studies, the primary aim is to demonstrate the feasibility of a patient-centered navigation model (often called "*Patientenlotsenmodell*") for stroke and lung cancer patients. In addition, initial estimates of the efficacy of the navigation intervention related to health-related quality of life, satisfaction with care, health literacy, utilization of care services, inpatient hospitalizations, mortality and cost-effectiveness as secondary endpoints will be made.<br><br>Research methods include quantitative and qualitative investigations of feasibility and efficacy parameters, as well as secondary data analyses with data from a health insurance.<br>The navigation model to be implemented was developed in a data-driven manner in a first project phase (ethics application numbers EA2/095/17 and EA2/122/19) and aims at supporting patients with the usually age-associated diseases lung cancer and stroke along their care pathway according to their individual situation, preferences and wishes to use the care services appropriate for them.<br>The intervention includes patients and their caregivers in the regions of Berlin and Brandenburg as target groups.

	Hypothesis: The intervention of a patient-oriented navigation model is feasible in terms of its acceptance, need and practicability in everyday care. To this end, we conduct a comprehensive process evaluation. Primary quantitative feasibility endpoints are the following criteria:<br>• 70% of intervention participants take part in the initial in-person interview with the navigator.<br>AND<br>• Less than 40% of intervention participants drop out of the intervention before the end of the 1-year period (excluding medical or logistical reasons).<br>In the case of successfully demonstrated feasibility, the efficacy of the intervention will be tested in relation to one of the patient-reported outcomes studied (e.g., health-related quality of life) as an additional primary outcome. (Authors' comment: defined to outcome 'satisfaction with care' in study registration prior to recruitment start)<br><br>Secondary endpoints of the study are health-related quality of life, satisfaction with care, health literacy, utilization of care, inpatient hospitalization, mortality and cost-effectiveness.<br>These will be collected from participants in the RCTs as well as participants in the cohort (patients who do not want a navigation intervention) at the same assessment time points.
3. Explanation of the relevance of the study	Studies (mainly from North America) show that patient navigation models can have positive effects on the care (e.g., faster start of treatment, improved adherence, and reduced hospitalization) of patients. Demographic change in Germany and the associated increase in the number of patients with age-related diseases and multimorbidity pose a major challenge to the German healthcare system. Patients often face difficulties in organizing complex care due to the high fragmentation of the

	health care system. This is especially true for vulnerable patient groups without caregivers or without existing social support and for older patients and patients with several medical conditions (multimorbidity). Various indication-specific pilot projects are currently being investigated in Germany. The evidence regarding these programs is not yet sufficient and evaluations are not focused on the aspects of feasibility and implementation of the interventions in the real health care setting. Furthermore, many of these programs are developed rather "expert"- and less patient-oriented (in the sense of an explicit consideration of individual wishes and preferences in the care), and a documentation and evaluation of the accessibility of vulnerable target groups is not given.<br>In order to develop a patient-oriented navigation model based on data, we first investigated barriers to care in two prototypical age-associated diseases, lung cancer and stroke, with a focus on the patient perspective in a first project phase. In addition, regional support offers for patients and their caregivers in the Berlin and Brandenburg region were identified and clearly compiled, and investigations were conducted to identify vulnerable patient groups. Based on these results, core components of the navigation model were defined. In summary, the navigator should be available to lung cancer and stroke patients and, if necessary, their caregivers as a constant and long-term contact person and support them with administrative and bureaucratic barriers as well as by referring them to support services. While the proactive approach and identification of patients with support needs should take place in the clinical and inpatient setting, the function and tasks of the navigator focus on the outpatient care phase in the case of support needs in the new disease-related life situation.<br>Investigating the feasibility of this model will provide insights into whether and how

	such a care model can be implemented in a real care setting. Is patient navigation feasible in a real care setting and will patients with special support needs be reached via the planned access strategies and at the appropriate time? First estimates of effectiveness and cost-effectiveness will additionally provide important insights for a possible future implementation of patient navigators/guides in standard care.
4. Which of the following provisions apply<br>a. Medical Devices Act according to § 23b MPG - exception of clinical examination<br>b. Radiation Protection Act and Radiation Protection Ordinance<br>c. Genetic Diagnostics Act<br>d. Data protection laws:<br>-Concrete specification of the data protection responsible body to comply with data protection law (for Charité =EU Data Protection Regulation (DSGVO), Berlin Data Protection Act - BlnDSG).<br>-If applicable, according to the group of participants additional state data protection laws to be observed or BDSG	EU General Data Protection Regulation (DSGVO),<br>Berlin Data Protection Act - BlnDSG<br>Brandenburg Data Protection Act - BbgDSG
5. If applicable: Designation and characterization of the test products	Patient-oriented navigation program for patients with lung cancer and stroke
6. Significant results of the preclinical tests or reasons for not carrying out of the same	Not applicable. No investigation according to German Medicines Act (AMG).
7. Essential content and results of the previous studies/applications of the products to be tested in the study	In the first phase of the project, the development of the patient-centered navigation program was supported by investigations on barriers in the care paths from the perspective of patients and care experts, by compiling existing support resources in the Berlin and Brandenburg region, and by conducting studies on vulnerable patients who do not receive optimal care.<br>The main findings of these studies were:<br>- Care processes are adapted to institutional needs and along bureaucratic structures of

	care institutions (health insurance, hospitals, outpatient care). This leads to a high burden for patients in the organization of care.<br>- Patients without social support are particularly vulnerable.<br>- Support services are available in the Berlin and Brandenburg region for many needs (see also the brochures with support services at https://navicare.berlin/de/ressourcen-fuer-patienten/). However, patients and, in some cases, healthcare providers are not aware of the existence of these services or it takes a lot of time and effort to find them.<br>- Patients often experience barriers in the organization of their care in the outpatient setting.<br>- The care of lung cancer patients in the inpatient setting is largely in accordance with the guidelines.
8. Description of the planned measures/examination methods and any deviations from the measures/examination's customary in in medical practice<br>(What is "routine", what will be done differently in the study?)<br><br>If validated questionnaires used in the study, please state the name of the questionnaires and where they have been published (references). Please attach non-validated questionnaires.	The feasibility and effectiveness of a patient-oriented patient navigation intervention will be investigated within the framework of a mixed-methods study in the design of a two-arm, controlled, randomized study with a parallel-running cohort study. Surveys will take place through quantitative interviews and data collection, qualitative investigations, and secondary data analyses of routine SHI data (see figure 2).<br><br>Recruitment:<br>Study participation is possible for patients and, under certain conditions, also for caregivers (caregivers are persons from the family as well as other persons close to the patient such as friends or neighbors who are entrusted with the organization of care). Caregivers can participate in the study if they are the legal guardians of a patient.<br>The study participants will be approached, informed and enrolled by the study staff in the recruitment centers (Stroke Units CCM/CVK (and intensive care unit – amendment from August 2021), Asklepios

	Klinikum Brandenburg (Amendment from August 2021: Changed to Klinik Hennigsdorf due to chance of cooperating physician), Lung Tumor Outpatient Clinic CVK and the Municipal Hospital Brandenburg). In addition, information material will be displayed on the wards that treat stroke and lung cancer patients. This will give interested persons the opportunity to actively contact for the study participation themselves.<br>Participation of informed patients:<br>The patient is directly approached for participation in the study. Upon inclusion, he/she declares his/her consent to the collection of the basic medical data, the questionnaire surveys, the collection of his/her health insurance data and, if necessary, qualitative data.<br>As a further option, the patient's family member can be included in the study as an additional study participant. Here, the patient declares his/her consent to the collection of his/her basic medical data and health insurance data as well as the consent that the caregiver can use the navigation in his/her interest. The caregiver declares his/her consent to answer the questionnaire surveys and, if necessary, qualitative data. Patient and caregiver can use the navigation.<br>Participation of patients who are not capable of giving informed consent:<br>If the patient himself/herself is incapable of giving informed consent and is legally represented by his/her caregiver, it is possible for the caregiver to participate in the study. In this case, the caregiver gives consent for the patient to have his/her basic medical data and health insurance data collected. For himself/herself, the caregiver declares his/her permission to answer the questionnaire surveys and, if necessary, qualitative data.<br>Participants will be randomized to the intervention arm or the control arm after giving informed consent.

	Randomization<br>Randomization will be performed within each patient group (stroke / lung cancer) stratified by center (Berlin, Brandenburg) and after inclusion of patient or family member as block randomization with changing block length in a ratio of 1:1. After inclusion of the patient by the study nurse, randomization will be performed by the Institute of Biometry and Clinical Epidemiology of the Charité. The randomization list is compiled by the biometricians involved. After randomization, the group membership will be reported back to the respective study nurses.<br><br>Comparison groups:<br>- Intervention group: Receives the navigation intervention described below for 1 year from initial interview with Navigator. After randomization, participant is informed about group allocation and receives contact information of the navigator.<br>- Control group: Does not receive support from the navigator. After randomization, the participant will be informed about the group allocation and will receive the brochure with support services for people affected by stroke or lung cancer and their caregivers (see pdf version here: https://navicare.berlin/de/ressourcen-fuer-patienten/).<br>Patients in both groups can utilize all standard care services.<br><br>Patients who decline randomization in the RCT will have the opportunity to participate in a parallel cohort survey.<br><br>Patients who meet the inclusion criteria but do not wish to participate in any of the study arms will be asked if they would be willing to provide their reasons. They can then fill out a "refuser questionnaire" or communicate their reasons to the study nurse, who will then fill out the questionnaire. Refuser questionnaires can

	be dropped anonymously in a box on the wards or given to the Study Nurse. They will be evaluated completely anonymously.<br><br><br>Furthermore, the total number of patients per visit at a recruitment site as well as baseline data (age, gender, ICD-10 code, severity/tumor stage, comorbidities) will be recorded anonymously for all patients via the hospital documentation system in order to compare participating and non-participating patients based on these parameters.<br><br>Using participant observation of the recruitment process as well as expert interviews with study nurses by a research associate of the project, the recruitment process will be flanked to identify potential barriers created by the selected settings (recruitment centers) for inclusion in a navigation model.<br><br>Intervention:<br>"Patient-Centered Navigation Program for Stroke/Lung Cancer Patients."<br>The navigator (Lotse) has a professional background in social work (alternatively nursing, case management, or comparable training) and will be further trained according to his/her previous experience in areas of patient-centered care, communication, existing support services for patients and disease-specific aspects of the studied diseases lung cancer and stroke. The training is modular and will be conducted on two consecutive days. The Institute of Social Medicine and Epidemiology at the Brandenburg Medical School is responsible for the training. Together with professional partners from the care sector (social workers, psychooncologists, physicians, communication scientists), the navigators are introduced to communication, existing services, barriers to patient-oriented care and their networking activities.

	For the navigation, the navigator will contact the recruited study participants of the intervention arm (up to 4 weeks after inclusion) or will be actively contacted by the participants in case of prior need. The navigator conducts an initial and personal interview with the study participants to identify the individual situation of the study participant and possible pre-existing support needs of the participants, as well as to establish the preferences of the study participants for the further form of interaction and communication. The location of the meeting is determined according to the needs of the study participant(s). This initial meeting will be face-to-face by default (Amendment from August 2021: possibility of video consultation due to pandemic situation) and planned to be of sufficient duration to identify the needs of the study participants and to agree on an individual plan of communication with the study participants as well as the initial frequency of contact. Independently of this, the navigators offer regular telephone consultation hours during which study participants can contact them. Navigation is planned for a duration of 1 year per study participant and the minimum frequency of contact between navigator and study participant is once every 3 months.<br><br>Tasks of navigation:<br>(a) Provide information to support daily living and further support options, such as counseling services, psychosocial and other support options.<br>(b) Help with application formalities, such as applying for a disability certificate or degree of care and rehabilitation measures.<br>(c) If necessary, establishing contact with (specialist) doctors, therapists and other support and care facilities.<br><br>The navigator will document the navigation process in detail with the study participants to obtain data for selected outcomes to

	investigate the feasibility of the navigation model.<br><br>The navigators have weekly team meetings for mutual support and information exchange. These meetings are accompanied by participant observation by a researcher. In addition, expert interviews with the navigators will be conducted as well as guideline-based interviews with study participants to capture how the navigators support study participants and how this is experienced by study participants and navigators.<br>This serves the purpose of process evaluation as well as the identification of possible needs for change in the navigation model.<br><br>Other data collection methods<br>a. Data collection RCT<br>Questionnaire Assessments:<br>To examine the effectiveness of the intervention, both groups will receive a baseline assessment on the day of study enrollment (if this is not feasible in individual cases, it may be conducted in the period up to 4 weeks after study inclusion). Follow-up interviews will take place 4 months, 7 months and 13 months after inclusion. (Amendment from August 2021 and January 2022: Vital status of patients is accessed from the registration office prior to follow-up and after study completion to identify lost-to-follow-up participants)<br><br>Questionnaires can be answered in writing by the study participant or with the help of caregivers or study staff if needed.<br><br>Secondary data analysis<br>The AOK Nordost provides pseudonymized routine data for all participants of the RCT who are insured with the AOK Nordost. These data contain information on individual treatment and care data, frequency of utilization of health care services, and data on associated costs. The

	data cover the period of the intervention and the previous year (25 months in total). The total data set will be made available about six months after completion of the intervention (last patient out). A partial data set will be made available approximately six months after the last participant's inclusion.<br><br>b. Data Collection Cohort Study<br>The assessment timing and content of the surveys in the cohort study will parallel the assessments of the RCT with: Baseline survey at inclusion, follow-up surveys at 4 months, 7 months, and 13 months.<br>In addition, guideline-based interviews will be conducted with participants in the cohort study to learn how they navigate their care.
9. Evaluation and consideration of foreseeable risks and disadvantages of study participation versus expected benefits for the study participants and in the future sick persons (benefit-risk assessment)	RCT<br>The current expected risks and disadvantages of participating in the RCT are:<br>- Feeling overwhelmed by interaction with navigator and study staff during assessment interviews in an emotionally stressful phase of illness.<br>- Feeling of disappointment in case of randomization into control arm of the study without care by navigator (no blinding possible).<br>- Time required for baseline and follow-up interviews of max. 1 hour.<br>These potential risks are offset by the presumed benefits of the navigation program for participants in the intervention arm. Navigation can reduce uncertainties and barriers in the patient's course of care in the short term. Participants in the control group also receive support in the form of the brochure with support services for stroke/lung cancer patients, which can provide orientation.<br>Long-term benefit is the gain of knowledge about the feasibility and effectiveness of the patient navigation model, which in the

	long term can contribute to the implementation of navigation services in standard care and thus to the reduction of barriers in care coordination in the German care landscape.<br><br>Cohort survey<br>The surveys at baseline and follow-up time points take a maximum of 1 hour per survey time point. This contrasts with the long-term gain in knowledge about the feasibility and need for navigation in the patient cohorts studied. This justifies the time required for the surveys.<br><br>Interviews with study participants:<br>The time frame for the interviews with the study participants depends on the narrative style and the narrative needs. As a rule, such interviews last about 60 minutes. The interviews may lead the study participants to recall unpleasant situations or to come to terms with their disease and its care. This can have both positive and negative consequences for them. Many study participants report that it was a relief for them "to be allowed to tell their story for once" and thus to experience connections of meaning in what had happened (literature: Dvorak F. Krankheitserfahrungen erzählen. Evaluation of participation in a patient-oriented health website from the perspective of the interviewees. Freiburg: Institute of Psychology; 2010). In contrast, there is the possibility that study participants only become aware of negative experiences and emotions as well as limitations through the interview. Very experienced researchers trained in interviewing conduct the interviews. If necessary, they consult the psychooncology department of the CCCC for supervision or refer the interview partners to the psychooncology department of the CCCC. Often, there is still a need after the interview. In this case, the study participants are given contact details for psychological support.

	Expert interviews with study nurses and navigators:<br>The interviews with the study nurses about their experiences with the recruitment process are time-consuming (approx. 30-45 min. per interview). Under certain circumstances, the interviews can lead to feelings of frustration and anger if they associate rather negative experiences with them. On the other hand, telling and reflecting on them can also be helpful to better process such experiences. For the implementation of the navigation model, it is crucial to find out whether the chosen settings are suitable for recruitment (and thus for the inclusion of participants in the navigation program). Therefore, the time spent on interviews is justified. The study nurses will be professionally accompanied by the Psychooncology Department of the CCCC, if necessary, to provide psychological support.<br><br>The interviews of the navigators are time-consuming (approx. 30-45 min. per interview). The interviews can lead to emotional stress for the navigators, for example if they remember unpleasant situations and feelings such as helplessness (e.g., if the navigators experience that they cannot help the study participants), excessive demands or frustration are evoked in them. In addition to the weekly team meeting, which is set up as a peer support, there is also the possibility to receive professional support from the psychooncology department of the CCCC if needed. The interviews are often experienced as helpful in strengthening self-reflection and resolving existing issues/dissatisfactions. The conversations and interview transcripts are shared with the project management only in an aggregated form. The principal does not have access to the original data of the Navigators, the recruitment process, the team meetings or the interviews with Study

	Nurses. Only the results are discussed with the project management.<br>Asking the navigators about the feasibility/feasibility of the navigation program and their experiences as providers of navigation services is a central component of the evaluation of the intervention and therefore justifies the effort and potential risks.<br><br>Participant observation:<br>Participant observation by a research assistant on the project during the recruitment phase will evaluate whether the settings chosen are appropriate for recruiting participants in a navigation program and how the navigation program is presented and received. Under certain circumstances, the study nurses may not feel quite as free in their work as they would without observation, although this risk is attempted to be minimized by having the participant observation conducted by a project staff member who is part of the team (and not by a stranger).<br>In addition, the regularly held team meetings of the navigators are accompanied by participant observation by a research assistant of the project. Under certain circumstances, the navigators might feel somewhat inhibited by the presence of the observing person, but this is a researcher of the project who is known to the navigators, which should minimize this risk.<br>Through the participant observation of the recruitment process as well as the team meetings of the navigators, important conclusions should be drawn regarding the implementation and feasibility of the navigation program. This justifies the effort and the risks.
a. Medical benefit to be examined for the study participants (individual benefit for the individual patient)	Benefits of patient navigation in terms of improvement of health-related quality of life or satisfaction with care will be investigated within the study.<br>In addition, previous studies from the international context have shown that

	patient navigation models can also have positive effects on care coordination as well as the frequency of hospitalizations of patients, which will be considered in the secondary data analyses with health insurance data.<br>Participants in the intervention arm can therefore benefit from the navigator, which is intended to support the reduction of barriers in personal care coordination and organization and thus contributes to a better integration of complex follow-up care. This can lead to a reduction in anxiety, stress, and faster and more targeted access to care. The participants in the control group also receive assistance in the form of a brochure with support services, which can lead to better knowledge of the support services and thus to a reduction in questions and barriers regarding the disease.<br>During the interviews with study participants, talking about their own experiences can be helpful for study participants to process what they have experienced. For study nurses and navigators, talking about their own experiences in connection with their own work in the context of the navigation program can also serve as self-reflection and thus be helpful. The reflection of the observations of the participant observation can also be helpful for study nurses and navigators to reflect on and process their own experiences and to initiate improvements for their own work.
9b. Medical benefit to be examined for persons with future disease (group benefit)	The aim of the study is to investigate the feasibility (and effectiveness) of a patient-oriented patient navigation model for patients with age-associated diseases (using the two prototypical diseases stroke and lung cancer as examples). If successful, the study may provide important insights for the implementation of such a navigation/guiding intervention in the German health care setting. In the long term, this can lead to an improvement in the organization of care processes and, as a

	secondary effect, to generally better networking and awareness of regional care and support services, which could lead to an improved use of these services by patients and their caregivers in case of need.
9c. Risks and burdens for study participants (list all in detail)	RCT<br>Participation in the RCT of the study may lead to feelings of being overwhelmed by multiple follow-up interviews.<br>In addition, it may lead to feelings of disappointment and being left alone if the study participant is randomized to the control group without care from the navigator.<br><br>Cohort<br>Participation in the study RCT may lead to feelings of being overwhelmed by multiple follow-up interviews.<br><br>Interviews with study participants:<br>Interviews may lead to study participants recalling uncomfortable situations or being confronted about their illness and care.<br><br>Secondary data analysis<br>The retrospective analysis of the pseudonymized health insurance data of the AOK-insured participants does not represent a risk or burden for them.
10. Risk control measures	Participation in all parts of the study is voluntary. Participants are informed that they may discontinue their participation in the study at any time without giving reasons. All data collected will be analyzed and reported in pseudonymous form.<br><br>The study staff and the navigators are professionally trained to recognize stress on the part of the study participants.<br>Support may also be needed after the navigation intervention/study participation. Therefore, patients and their caregivers are given contact information for support. This information is available in the study information.

	For lung cancer patients:<br>- CCCC hotline:<br>- Telephone number of the head of psychooncology at the CCCC [deleted for privacy reasons for the PLOS ONE protocol publication]<br><br>For stroke patients:<br>- Advice hotline of the stroke service point: [deleted for privacy reasons for the PLOS ONE protocol publication]<br><br>In addition, these and other contact addresses are listed in the brochure with support services.
11. Termination criteria	- Participant withdraws consent<br>- Study personnel notice a psychological burden that makes it necessary to discontinue the study. This is then done in consultation with the psychooncology department of the CCCC or the medical contact persons in neurology.
12. Number, age, and sex of persons affected	Planned case numbers:<br>1. Stroke.<br>A total of 685 stroke patients and, if applicable, caregivers (male/female/diverse, age ≥18 years) will be included in the overall study.<br>These will be divided among the study parts as follows:<br>a. RCT<br>460 stroke patients (and caregivers, if applicable).<br><br>b. Cohort study<br>225 stroke patients,<br><br>c. Qualitative study<br>Study participants from RCT and cohort study<br>(in addition, 3 study nurses/navigators (male/female/diverse) aged 18 years and older)<br>d. Secondary data analysis<br>Approximately 165 stroke patients from RCT (depending on the actual AOK insured proportion in the RCT),

	2. lung cancer<br>A total of 195 lung cancer patients and, if applicable, caregivers<br>(male/female/diverse, age ≥18 years) will be included in the overall study.<br>These will be divided among the study parts as follows:<br>a. RCT<br>120 lung cancer patients (and caregivers, if applicable).<br><br>b. Cohort study<br>75 lung cancer patients<br><br>c. Qualitative study<br>Study participants from RCT and cohort study<br>(in addition, 3 study nurses/navigators (male/female/diverse) aged 18 years and older)<br><br>d. Secondary data analysis<br>Approx. 43 lung cancer patients from RCT (depending on the actual AOK-insured proportion in the RCT)
13. Biometric planning with indication of the statistical methodology, including justification of the number of cases. Indication of the statistician (if advice is given by the Institute of Biometry of the Charité, a signature must be included)	Biometric Planning:<br>Biometric calculations were calculated based on the two defined primary feasibility outcomes for determining feasibility. These are as follows:<br>"The intervention is feasible if:<br>(1) At least 70% of patients* in the intervention arm participated in at least one initial face-to-face navigator session.<br>AND<br>(2) The dropout rate of the intervention arm of the RCT is less than 40% (here, dropouts are defined as study participants who drop out of the intervention for reasons other than those that physically prevent the patient*s/caregivers from participating. E.g., move out of catchment area, deterioration in general health, long-term hospitalization, move to nursing home or hospice, death).<br><br>Explanations in the individual study sections:

	1. Stroke<br>a. RCT<br>For the duration of the recruitment period, we expect a population of about 1850 stroke patients at the three planned recruitment sites (1100 patients at Charité Universitätsmedizin Berlin stroke units at the sites Mitte and Virchow Klinikum, 750 patients at Asklepios Fachklinikum Brandenburg (Amendment from August 2021: Changed to Klinik Hennigsdorf due to change of cooperating physician)) based on data from previous years. We assume that 70% of these patients can be approached by the study personnel for participation in the study. Of the patients approached, we expect a recruitment rate (depending on recruitment site) of 30-50%. Based on these assumptions, we expect 460 stroke patients (and their caregivers) to participate.<br>Based on the feasibility criteria defined above, the statistical planning is as follows: If 460 stroke patients are included in the study, and 230 (50%) of the patients* are randomized to the intervention arm, we assume that at least 95% (n=219) will still have survived to receive patient navigation at 4 weeks. If 166 (75.8%) or more of these 219 patients* receive the initial navigation session, the first feasibility criterion is met, as the 95% confidence interval of this proportion will not be less than 70% (95%CI: 70.1%-81.5%). We additionally assume that of these 230 patients (intervention arm), 85% (n=196) survived after one year. If of these 196 patients, 65 (33.2%) or less are "lost-to-follow-up", the second feasibility criterion will be met, as the 95% confidence interval of this proportion will be less than 40% (95% CI: 26.6%-39.8%).<br>The feasibility of the study is considered successful if both criteria are achieved.<br><br>In case of successfully demonstrated feasibility, we additionally test the efficacy of the intervention in the study at the two-sided significance level α=0.05 using

	ANCOVA, adjusted for baseline measurement of a selected patient-reported outcome (e.g., health-related quality of life (Authors' comment: defined to outcome 'satisfaction with care' in study registration prior to recruitment start)) and adjusted for the stratification variables of randomization (hierarchical testing). All primary questions are analyzed confirmatory manner in the "full analysis set." In case of missing values and assuming missing at random, multiple imputation models are used to estimate the missing values.<br><br>In addition to the analyses of primary outcomes described at the outset, additional feasibility criteria will be evaluated descriptively/exploratively as secondary outcomes and patient-reported outcomes (see Table 1-3) will be compared between intervention arms in univariate analyses and multiple regression models. Exploratory subgroup analyses will be conducted to examine differences in feasibility and effectiveness in relation to predefined comparators.<br><br>b. Cohort Study<br>We expect the cohort study to have a participation rate of 30% of approached patients who decline to participate in the RCT. This results in 225 stroke patients in the cohort study.<br><br>Collected data of patient-reported outcomes will be analyzed exploratively/descriptively and compared longitudinally.<br><br>c. Process evaluation<br>Process evaluation, including qualitative interviews with study participants, will not require biometric design; rather, sampling will be used to map experiences with the intervention until study results are considered saturated. Experience from other qualitative studies suggests that the

	total number of 30-40 interviews will provide a good picture of the different experiences with the intervention. Selection criteria in each case are age, gender, severity of illness, family status (living alone vs. living with partner), presence vs. absence of social support, satisfaction vs. dissatisfaction with navigation; difficulties with navigation.<br><br>d. Secondary data analysis<br>Based on the assumed 460 patients included in the RCT and the average proportion of AOK-insured patients of approximately 36% (as of 2018) in the total population of Germany, we expect a case number of 165 for the secondary data analysis of insurance data.<br>The data will be analyzed exploratively/descriptively and compared longitudinally. The usually non-normally distributed cost data will be accounted for in the statistical analyses with appropriate procedures.<br><br>2. Lung cancer<br>a. RCT<br>For the duration of the recruitment period, we expect a population of approximately 550 lung cancer patients at the planned recruitment sites (Charité Universitätsmedizin Lungentumorambulanz at the Virchow Klinikum site, 50 patients at the Städtisches Klinikum Brandenburg). We assume that 70% of these patients can be approached by the study personnel for participation in the study. Of the patients approached, we expect a recruitment rate (depending on location) of 30-50%. Based on these assumptions, we expect 120 lung cancer patients (and their caregivers) to participate.<br>Regarding the feasibility criteria defined above, the statistical planning is as follows: If 120 lung cancer patients are enrolled in the study, and 60 (50%) of the patients* are randomized to the intervention arm, we assume that at least 95% (n=57) will still

	have survived to receive patient navigation at 4 weeks. If 46 (80%) or more of these 57 patients* receive the initial navigation session, the first feasibility criterion is met, as the 95% confidence interval of this proportion will not be less than 70% (95%CI: 70.5%-90.9%). We additionally assume that of these 57 patients (intervention arm), 75% (n=43) survived after one year. If of these 43 patients, 11 (25.6%) or less are lost-to-follow-up, the second feasibility criterion will be met, as the 95% confidence interval of this proportion will be less than 40% (95% CI: 12.5%-38.6%).The feasibility of the study is considered successful if both criteria are achieved.<br><br>Analogous to the stroke study, if feasibility is successfully demonstrated, the efficacy of the intervention with respect to a selected patient-reported outcome (e.g., health-related quality of life (Authors' comment: defined to outcome 'satisfaction with care' in study registration prior to recruitment start)) is additionally tested as a primary question at the two-sided significance level of α =0.05 (hierarchical testing). If feasibility cannot be demonstrated, all other outcomes will be analyzed secondarily in an exploratory manner.<br><br>In addition to the analyses of primary outcomes described at the outset, additional feasibility criteria will be evaluated descriptively/exploratively as secondary outcomes and patient-reported outcomes will be compared between intervention arms in univariate analyses and multiple regression models. Exploratory subgroup analyses will be conducted to examine differences in feasibility and effectiveness related to predefined comparators.<br><br>b. Cohort Study<br>We expect the cohort study to have a participation rate of 30% of approached

	patients* who decline to participate in the RCT. This results in a sample size of 75 lung cancer patients in the cohort study.<br>Collected data of patient-reported outcomes will be analyzed exploratively/descriptively and compared longitudinally.<br><br>c. Process evaluation<br>Process evaluation, including qualitative interviews with study participants, will not require biometric planning; rather, sampling will be used to map experiences with the intervention until study results are considered saturated. Experience from other qualitative studies suggests that the total number of 30-40 interviews will provide a good picture of the different experiences with the intervention. Selection criteria in each case are age, gender, severity of illness, family status (living alone vs. living with partner), presence vs. absence of social support, satisfaction vs. dissatisfaction with navigation; difficulties with navigation.<br><br>d. Secondary data analysis<br>Based on the assumed 120 patients included in the RCT and the average proportion of AOK-insured patients of about 36% (as of 2018) in the total population of Berlin and Brandenburg, we expect a case number of 43 for the secondary data analysis of health insurance data.<br>The data will be analyzed exploratively/descriptively and compared longitudinally. The usually non-normally distributed cost data will be accounted for in the statistical analyses with appropriate procedures.
14a. Statement and, if necessary, explanation of the inclusion and exclusion criteria	Inclusion/exclusion criteria:<br>1. Stroke<br>a. RCT<br>All stroke patients who are treated in the recruitment centers and during the recruitment period of 1 year, as well as their caregivers, can be included in the

	study. Due to the examination in the real-world care setting and environment, no comprehensive exclusion of patients with comorbidities should be carried out. Exceptions are listed in the exclusion criteria.<br>Inclusion Criteria:<br>• Confirmed diagnosis of stroke/TIA (ICD-10 codes: G45.x, I60.x, I61.x, I63.x, I64.x H34.x (amendment from August 2021), H47.0 (amendment from May 2022)<br>• Caregivers of a patient diagnosed with stroke (with the consent of the patient or existing legal representation)<br>• Age: ≥18 years<br>• resident in Berlin and Brandenburg<br><br>Exclusion criteria:<br>• Nursing home resident at the time of inclusion<br>• Patients without relatives who are unable to give informed consent and for whom there is no existing legal care by the caregivers<br>• Dementia (here the inclusion of caregivers can be possible)<br>• Language barrier (here the inclusion of caregivers can be possible)<br><br>b. cohort study<br>As a, but no participation of caregivers planned<br><br>c. qualitative study<br>Study participants from a and b.<br><br>d. Secondary Data Analysis<br>All RCT participants who are insured with AOK Nordost are included in this part of the study.<br>Inclusion Criteria:<br>• Participation in the RCT<br>• Insured with AOK Nordost<br><br>2. Lung Cancer<br>a. RCT<br>All lung cancer patients who are treated in the recruitment centers and during the

	recruitment period of 1 year and their caregivers can be included in the study. Due to the examination in the real-world care setting and environment, no comprehensive exclusion of patients with comorbidities should be carried out. Exceptions are listed in the exclusion criteria.<br>Inclusion Criteria:<br>• Confirmed diagnosis of lung cancer (ICD-10 codes: C34.1, C34.2, C34.3, C34.8, C34.9, C97)<br>• Caregivers of a patient diagnosed with lung cancer (with the consent of the patient or existing legal representation)<br>• Age: ≥18 years<br>• resident in Berlin and Brandenburg<br><br>Exclusion criteria:<br>• Nursing home resident at the time of inclusion<br>• Patient without relatives who are not able to give informed consent and for whom there is no existing legal care by the caregiver<br>• Dementia (here the inclusion of caregivers can be possible)<br>• Language barrier (here the inclusion of caregivers can be possible)<br><br>b. cohort study<br>As a, but no participation by caregivers planned.<br><br>c. qualitative study<br>Study participants from a and b.<br><br>d. Secondary Data Analysis<br>In this part of the study, all RCT participants who are insured with the AOK Nordost and have given their consent to the query of the GKV routine data are included.
14b. Study information (who gives this verbally and in writing and indication of how much time remains between information and consent (written information as an annex)	Information is provided verbally by study personnel. In addition, the patient will receive written study information.<br>The patient or family member will be given sufficient time to clarify any unanswered

	questions. There is the possibility of study inclusion on the day of the information and, if desired, also the possibility of later contact with/by the study team and inclusion of the patient/caregiver. For this purpose, the patient receives an information flyer about the study, including contact information.<br>After the patient has been informed, he/she has the choice to participate in the RCT part or in the cohort part of the study.
14c. Informed consent (written form attached)	Consents and contact form
14d. If applicable, information and consent of the legal representative (if applicable, also description of the procedure for the establishment of a legal guardianship)	If the patient is under legal guardianship, the consent of the legal representative is obtained.
15. Measures for the recruitment of study participants (notice board? If applicable, reason for inclusion and explanation of therapeutic benefit for persons who are minors and/or incapable of giving consent.	Stroke:<br>For contacting and recruiting the study participants, the already existing cooperation with the Trial Team of the Center for Stroke Research (Head: Prof. Dr. Christian Nolte), the Clinics for Neurology of the Charité, as well as the Asklepios Klinikum Brandenburg (Amendment from August 2021: Changed to Klinik Hennigsdorf due to change of cooperating physician) and the Stroke Units of the Charité (and intensive care unit – amendment from August 2021)will be used.<br>After initial screening of eligible patients, patients are actively approached by the study personnel. In addition to verbal information, the patient and/or their caregiver also receive written study information.<br>Furthermore, flyers and, if possible, posters in the wards draw attention to the study. Here, the study is presented in a manner appropriate to the target group and the patient and/or caregivers are given the opportunity to contact the study coordinator by providing contact information.<br>Within the framework of this procedure, participants can be included in the RCT and the cohort study.

	(Amendment from January 2022: due to severe pandemic situation with access restrictions, recruitment is handled by increased distribution of information material. And patients that couldn't be reached in the hospital are contacted via postal letter with study information and return envelope in collaboration with trial team of Center for stroke research. Furthermore, study enrollment of patients and caregivers that actively contact the study coordination for participation is made possible independent from recruitment site)<br><br>Lung Cancer:<br>The existing cooperation with the Charité Comprehensive Cancer Center, the lung cancer outpatient clinic of the Charité and the Brandenburg Municipal Hospital will be used to establish contact and recruit study participants.<br>After initial screening of the eligible patients, the patients are actively approached by the study staff in the outpatient clinic or ward. In addition to verbal information, the patient and/or their caregiver also receive written study information.<br>In addition, flyers and, if possible, notices and displays at the recruitment sites will draw attention to the study. Here, the study is presented in a manner appropriate to the target group and the possibility of contact by the patients and/or caregivers is made possible by providing contact information for the study coordination.<br>Within the framework of this procedure, participants can be included in the RCT and the cohort study.
16. If applicable, reason for inclusion and explanation of therapeutic benefit for persons who are minors and/or incapable of giving consent.	No minors will be included in the study.
17. Relationship between study participant and study physician (is the study physician also the treating physician?)	Not applicable.

18. Statement on the inclusion of persons who may be dependent on the sponsor	Does not apply. No sponsor-dependent subjects will be included in the study.
19. Measures that allow determination of whether a study participant is participating in more than one study at the same time or before the end of a period specified in the previous study. Is participation in more than one study possible?	The possibility of participation in several studies is possible in general.<br>The simultaneous participation in other studies is excluded:<br>- If the studies are intervention studies for the evaluation of new forms of care or if the study significantly changes the standardized care process.<br><br>By agreement with the coordinating institutions for study participation of the study centers, this will be checked with the patients after consent has been obtained. In case of doubt, a case-by-case assessment is carried out, which takes into account the type and extent of an additional study participation.
20. If applicable: remuneration or reimbursement of study participants (amount, what should be paid for?)	Participants RCT and cohort:<br>No remuneration planned, unless participants conduct a qualitative interview (duration approx. 1 hour) in addition to the usual assessments of the study. In that case, payment of an expense allowance of 25 euros is planned.
21. If applicable: plan for further treatment and medical care of the persons concerned after the end of the study	After completion of the study after 1 year, no further care by the patient navigator is planned and patients receive the usual measures of standard care.<br>Participants also receive the NAVICARE brochure with support services.
22. If applicable: insurance of the study participants (insurance confirmation and insurance conditions, insurer, scope of insurance, duration of insurance)	Covered by general public liability insurance of Charité and Brandenburg Medical School.
23. Documentation procedures:<br>- If applicable: reference to CRF forms<br>- specification of the data to be collected<br>- sample handling- storage/archiving (incl. deadlines)<br>- access to data and samples	A detailed table of the indicators to be studied and the resulting data to be collected is shown in Table 1-3.<br><br>Data collected for the evaluation of the navigation process are shown in Figure 3 as a draft of a possible survey structure. These data will be collected in writing by the navigator as part of their general

	documentation. Data for evaluation will be transmitted electronically by the navigator.<br><br>RCT and Cohort<br>CRF medical data will be extracted by study staff from study center medical information systems. Data on hospitalizations during the course of study participation will be obtained by querying discharge letters from hospitals.<br>Questionnaire data will be collected through written or, if needed, telephone interviews.<br>All data will be collected electronically through the REDCap system.<br>The electronically collected data are transferred to the REDCap server of the Berlin Institute for Health (BIH) via a network connection. Temporarily stored data on a mobile device is encrypted by the application. At regular intervals, a deletion of all local data of the application takes place. (See REDCap operating manual version 1.1 dated December 17, 2015, issued only by Charité IT security officers). There is a positive data protection vote for the REDCap system from Charité as well as Berlin Data Protection dated Jan. 13, 2016. Worldwide, the open-source REDCap system is used in more than 1792 institutions and 240000 studies/projects (www.project-redcap.org).<br><br>Access to identifying data is restricted to members of the study coordination team at the Institute of Public Health.<br>Data will be transmitted to and analyzed by other study staff members only in a pseudonymized manner.<br><br>Process evaluation:<br>Interviews with study participants:<br>Interviews with study participants will be digitally recorded using a voice recorder. The audio recordings will be stored on separately protected areas of the server of the Brandenburg Medical School, pseudonymized transcribed, analyzed with

	the analysis software MAXQDA® and stored for at least 10 years after the end of the study. The publication is done with pseudonymized data. Only authorized staff members of the project have access to the data.<br><br>Interviews with Study Nurses and Navigators:<br>The interviews with the study nurses and navigators of the project are digitally recorded with a voice recorder. The audio recordings are stored on separately protected areas of the server of the Brandenburg Medical School, transcribed pseudonymously, managed with the analysis software MAXQDA® and stored for at least 10 years after the end of the study. Publication is done with pseudonymized data. Only authorized staff members of the project have access to the data.<br><br>Participant observation:<br>During the participant observation, handwritten protocols will be prepared by the research assistant. These protocols are stored in locked cabinets at the Institute of Social Medicine and Epidemiology of the Brandenburg Medical School and are only accessible to authorized project staff. The handwritten protocols will subsequently be converted into digital documents. The digital documents are stored on separately protected servers at the Brandenburg Medical School. Only authorized personnel of the project have access to the data. Both the handwritten protocols and the digital documents will be stored for at least 10 years after the end of the study.<br><br>Routine data:<br>To secure the data, the following measures will be implemented - analogous to the first funding phase of CoreNAVI:<br>The pseudonymized insured data transmitted by the AOK Nordost will be stored encrypted on a server of the Charité. Data access is password-protected and only

	possible for selected staff members of the project team of the Institute of Medical Sociology and Rehabilitation Science.<br>According to good practice secondary data analysis (GPS) (Swart et al. 2015), the pseudonymized data will be stored encrypted for a period of 10 years. This serves to reconstruct and, if necessary, reproduce the analysis results at a later time.<br>Only members of the study coordination at the Institute of Public Health have access to identifying data.
24. If applicable: description of how the health status of healthy subjects is to be documented	Not applicable.
25. If applicable: methods of detecting, documenting and reporting adverse events (when, by whom and how?)	Not applicable.
26. Procedure for protecting the confidentiality of stored data, documents and, if applicable, samples, description of the pseudonymization or anonymization of data and samples of study participants (initials and date of birth are not permitted as a coding scheme!)<br>- Description of the separation of medical files, study documentation and allocation of personal data<br><br>- Naming of access rights including access to participant identification lists during and after the study<br>- Detailed specification of the procedures for transmission, encryption, restriction of processing (blocking) and deletion (including specification of the network structure used and servers used if applicable)<br>- If applicable, access to identifying data for legally authorized examiners (third parties) for the purpose of viewing the files required for this purpose.	All study participants receive a pseudonymized participant ID after study inclusion (randomization, if applicable), which does not contain any identifying characteristics. The creation of the participant ID is performed in an automated process.<br><br>All data will be recorded pseudonymously in the REDCap documentation system.<br><br>Original study documents (consents, questionnaires) are stored in lockable cabinets for 10 years. Here, identifiable documents are stored spatially separated. Access to identifying data is restricted to the principal investigator and persons directly authorized by the principal investigator within the study coordination of the Institute of Public Health.<br><br>In order to obtain the GKV routine data of the study participants of the RCT, an encrypted list with relevant identifying variables (insured person number, date of birth, study pseudonym) will be transmitted to the AOK. In this way, the AOK can identify the insured persons in the SHI routine data set and extract the data of the

	corresponding insured persons. The pseudonymization of the patient data of the AOK is ensured by the data owner (AOK Nordost) in compliance with data protection regulations by deleting the identifying variable (insurance number) in the routine dataset and replacing it with the study pseudonym known to the AOK. In addition, personal identifying characteristics contained in the data set (e.g. name, place of residence) are also deleted. Apart from the evaluation variables, the data set now only contains the study pseudonym. These insurance data are encrypted by the AOK Nordost and made available to the Charité via a data sharing point. Here, the data are stored on a password-protected server of the Charité. Only authorized project staff members have access to data. Since the data still have the study pseudonym at this point, a linkage with the primary data collected in the study (e.g. quality of life) can take place. Subsequently, the deletion of the study pseudonym takes place, so that the linked data can be regarded as de facto anonymous from this point on. In this form, linked data sets are made available to the evaluating institutions.
27. Declaration of compliance with data protection<br>- Assurance that all data collected and stored about the study participant will be treated confidentially (data secrecy and medical confidentiality).<br>- Assurance that the identifying data will only be accessible to the study director or to employees appointed by him.<br>- Declaration of compliance with data protection<br>- Measures to ensure that the data is transferred in a manner that does not allow third parties to establish a personal connection	The handling of the collected data is subject to the data protection laws of the responsible federal state Berlin (BlnDSG), Brandenburg (BbgDSG) and the data protection by the SGB X and the Data Protection Regulation (DSGVO). According to the standard of good scientific practice, the data will be stored for a period of 10 years. The data is treated confidentially and disclosure of the data to unauthorized third parties is excluded. To ensure confidentiality, all collected data are stored only on the servers of the Charité and the Brandenburg Medical School (for qualitative data) located in Germany. There will be no transfer of data abroad. The data are only accessible to the principal investigator and the authorized study personnel.

- Information on the possibilities for access, revocation, correction and deletion<br>- Measures to ensure the rights of the participants<br>- If transfers to non-EU countries are planned: Measures to ensure compliance with data protection (e.g. existence of an adequacy decision by the EU Commission or explicit consent of the study participants to such transfers).	Only the principal investigator and the authorized personnel from the study coordination at the Institute of Public Health have access to the identifying data. Thus, the rights of access, revocation, correction and deletion are assured.<br>The data are not traceable to individual persons for study personnel outside the study coordination at the Institute of Public Health.<br>The data protection concept will be coordinated with the data protection officers of Charité and AOK Nordost in parallel to the application.
28. Names and addresses of the institutions involved in the study as study centers or study laboratories, as well as the study directors and the study physicians - information on external service providers involved, including information on the possibility of accessing data	Priv.-Doz. Dr. Nina Rieckmann (Principal investigator), Institute of Public Health, Charité-Universitätsmedizin Berlin, Berlin (until July 2022)<br>Prof. Dr. Christine Holmberg (Principal investigator) Brandenburg Medical School Theodor Fontane, Brandenburg/Havel<br><br>Priv.-Doz. Dr. Dipl.-Psych. Ute Goerling, Charité Comprehensive Cancer Center, Charité-Universitätsmedizin Berlin, Berlin<br><br>Prof. Dr. Andreas Meisel, Center for Stroke Research Berlin, Department of Neurology, Charité-Universitätsmedizin Berlin, Berlin<br><br>Priv.-Doz. Dr. Susanne Schnitzer, Institute of Medical Sociology and Rehabilitation Science, Charité- Universitätsmedizin Berlin, Berlin<br><br>Priv.-Doz. Dr. Ulrike Grittner, Institute of Biometry and Clinical Epidemiology, Charité - Universitätsmedizin Berlin, Berlin<br><br>Prof. Dr. Thomas Reinhold<br>Institute of Social Medicine, Epidemiology and Health Economics<br>Charité- Universitätsmedizin Berlin, Berlin<br><br>Recruiting Study Centers:<br><br>Stroke:

	Department of Neurology Charité/Center for Stroke Research.<br>Charité- Universitätsmedizin Berlin<br>Prof. Dr. Andreas Meisel<br><br>Asklepios Specialist Hospital Brandenburg (Amendment from August 2021: Changed to Klinik Hennigsdorf due to change of cooperating physician)<br>Clinic for Neurology<br>Prof. Dr. Stephan Schreiber<br><br>Lung cancer:<br>Lung Tumor Outpatient Clinic<br>Charité Lung Cancer Center<br>Charité- Universitätsmedizin Berlin<br>Dr. med. Nikolaj Frost<br><br>Municipal Hospital Brandenburg<br>Center for Internal Medicine II<br>Prof. Dr. med. P. Markus Deckert<br><br>Scientific staff / researchers:<br>Hella Fügemann<br>Dr. Kathrin Gödde<br>Raphael Kohl
29. Information on the suitability of the trial site, in particular on the adequacy of the resources and facilities available there, as well as on the personnel available to conduct the clinical trial and on experience in conducting similar trials	The multiprofessional team of scientists (physicians, epidemiologists, anthropologists, psychologists, health scientists) at the Institute of Public Health has many years of comprehensive expertise and competence in planning, conducting and evaluating epidemiological and qualitative studies. Research focuses on cardiovascular and cancer diseases in the context of decision-making processes, health-related quality of life and patient-centered health services research.<br>The institutions involved in the study also have extensive and long-standing experience in planning and implementing research projects.
30. Agreement on access of the investigator/principal investigator to the data and the principles of publication.	The principal investigator has full access to the collected data. Publication of the study results in national and international peer review journals and in the form of presentations at congresses is planned. No

- Publications in a form that does not allow any conclusion on the person.	conclusions can be drawn about individuals when presenting the results.
31. Information on the funding of the study: source of funding (name and location) and amount of funding in €.<br>- If applicable, indication of the cost center for the ILV accounting of the fee.	Federal Ministry of Education and Research [Address and funding amount deleted for PLOS One publication]
