# Supplementary material for: Feasibility of a patient-oriented navigation programme for patients with lung cancer or stroke in Germany: Protocol of the CoreNAVI study
Source: PLoS One. 2023 Jun 29;18(6):e0287638. doi: 10.1371/journal.pone.0287638 (PMC10309639; doi:10.1371/journal.pone.0287638)
Supplement: S2 File — (PDF) [file pone.0287638.s003.pdf]

Haupttext zum Antrag auf Beratung durch die Ethikkommission (kommentierte Version):

Antrag auf Beratung durch die Ethikkommission zur Durchführung eines medizinisch-wissenschaftlichen Vorhabens, welches weder die klinische Prüfung eines Arzneimittels noch Medizinproduktes beinhaltet

Titel der Studie	CoreNAVI– Untersuchung der Machbarkeit eines patientenorientierten Navigationsmodells bei Schlaganfall und Lungenkrebs
1. Entscheidungen anderer Ethikkommissionen in derselben Sache	Nein
2. Gegenstand der Studie und ihre Ziele; Angabe der Hypothesen, getrennt in Haupt- und Sekundärhypothesen sowie der klinischen Parameter (primäre und sekundäre Endpunkte), anhand derer die Hypothesen geprüft werden sollen	Primäres Ziel:<br>Im Rahmen einer Mixed-Methods-Studie im Design von zwei zweiarmigen, randomisierten, kontrollierten Studien mit parallelen Kohortenstudien ist der Nachweis der **Machbarkeit eines patienten-orientierten Navigationsmodells (häufig auch Patientenlotsenmodell genannt) für Schlaganfall- und Lungenkrebspatient*innen das primäre Ziel**. Zudem sollen erste Schätzungen der **Wirksamkeit der Navigationsintervention bezogen auf die gesundheitsbezogene Lebensqualität, Zufriedenheit mit der Versorgung, Gesundheitskompetenz, Inanspruchnahme von Versorgungsleistungen, stationäre Krankenhausaufenthalte, Mortalität und Kosteneffektivität als sekundäre Endpunkte** erfolgen.<br><br>Erhebungsmethoden beinhalten quantitative und qualitative Untersuchungen der Machbarkeit und Wirksamkeitsparameter sowie Sekundärdatenanalysen von Abrechnungsdaten einer Krankenkasse<br>Das zu implementierende Navigationsmodell wurde in einer ersten Projektphase datengestützt entwickelt (Ethikantragsnummern EA2/095/17 und EA2/122/19) und hat das Ziel, Patient*innen mit den in der Regel altersassoziierten Erkrankungen Lungenkrebs und Schlaganfall entlang ihres Versorgungsweges entsprechend ihrer individuellen Situation, Präferenzen und Wünsche dabei zu unterstützen, die für sie angemessenen Versorgungsangebote in Anspruch zu nehmen.<br>Die Intervention schließt Patient*innen und ihre Angehörigen der Regionen Berlin und Brandenburg als Zielgruppen mit ein.<br><br><br>Hypothese: Die Intervention eines patienten-orientierten Navigationsmodells ist in Bezug auf seine Akzeptanz, Bedarf und Praktikabilität im Versorgungsalltag machbar. Dazu führen wir eine umfassende Prozessevaluation durch. **Primäre quantitative Machbarkeitsendpunkte** sind folgende Kriterien:<br>• 70% der Interventionsteilnehmer*innen nehmen das persönliche Eingangsgespräch mit dem/der Navigator*in in Anspruch.<br>UND

	• Weniger als 40% der Interventionsteilnehmer*innen brechen die Intervention vor Ende der Laufzeit von 1 Jahr ab (ausgenommen medizinische oder logistische Gründe).<br>Im Falle der erfolgreich nachgewiesenen Machbarkeit wird die Efficacy der Intervention in Bezug eines der untersuchten patientenberichteten Outcomes (z.B. gesundheitsbezogene Lebensqualität) als zusätzliches primäres Outcome getestet. (Anm. d. Autors: Hier wurde als Outcome ‘Zufriedenheit mit der Versorung’ im Rahmen der Studienregistrierung vor Rekrutierungsstart definiert)<br><br>**Sekundäre Endpunkte** der Studie sind gesundheitsbezogene Lebensqualität, Zufriedenheit mit der Versorgung, Gesundheitskompetenz, Inanspruchnahme von Versorgungsleistungen, stationäre Krankenhausaufenthalte, Mortalität und Kosteneffektivität.<br>Diese werden von Teilnehmenden der RCTs sowie den Teilnehmenden der Kohorte (Patient*innen, die keine Navigationsintervention wünschen) zu gleichen Messzeitpunkten erhoben.
3. Erläuterung der Bedeutung der Studie	Studien (vor allem aus dem nordamerikanischen Raum) zeigen, dass Patienten-Navigationsmodelle positive Effekte auf die Versorgung (z.B. schnellerer Beginn der Behandlung, verbesserte Adhärenz, geringere Hospitalisierung) von Patient*innen haben können. Durch den demographischen Wandel in Deutschland und eine damit verbundene steigende Zahl an Patient*innen mit altersbedingten Krankheiten und Multimorbidität wird das dt. Gesundheitssystem vor eine große Herausforderung gestellt. Für Patient*innen ergeben sich durch eine hohe Fragmentierung des Gesundheitssystems häufig Schwierigkeiten bei der Organisation der komplexen Versorgung. Dies gilt vor allem für vulnerable Patientengruppen ohne Angehörige bzw. ohne vorhandene soziale Unterstützung und bei älteren Patient*innen und bei Patient*innen mit mehreren Krankheitsbildern (Multimorbidität). Diverse indikationsspezifische Lotsenprojekte werden in Deutschland momentan untersucht. Die Evidenz bezüglich dieser Programme ist noch nicht ausreichend und Evaluationen sind nicht auf die Aspekte der Machbarkeit und der Implementierung der Interventionen im realen Versorgungsumfeld fokussiert. Ferner sind viele dieser Programme eher „Experten“- und weniger patientenorientiert (im Sinne einer expliziten Berücksichtigung individueller Wünsche und Präferenzen in der Versorgung) entwickelt, und eine Dokumentation und Evaluation der Erreichbarkeit vulnerabler Zielgruppen ist nicht gegeben.<br>Um datengestützt ein patienten-orientiertes Navigationsmodell zu entwickeln, haben wir in einer ersten Projektphase zunächst Barrieren in der Versorgung bei zwei prototypischen altersassoziierten Erkrankungen, Lungenkrebs und Schlaganfall, mit Fokus auf die Patientenperspektive untersucht.

	Darüber hinaus wurden regionale Unterstützungsmöglichkeiten für Patient*innen und Angehörige in der Region Berlin und Brandenburg identifiziert und übersichtlich zusammengestellt sowie Untersuchungen zur Identifikation von vulnerablen Patientengruppen durchgeführt. Anhand dieser Ergebnisse wurden Kernkomponenten des Navigationsmodells definiert. Zusammenfassend soll der/die Navigator*in den Lungenkrebs- und Schlaganfallpatient*innen sowie ggfls. ihren Angehörigen als konstante*r und langfristige*r Ansprechpartner*in zur Verfügung stehen und sie bei administrativen und bürokratischen Hürden sowie durch die Vermittlung an Unterstützungsangebote unterstützen. Während die **proaktive Ansprache** und Identifikation von Patient*innen mit Unterstützungsbedarf im klinischen und stationären Setting stattfinden soll, fokussieren sich Funktion und Aufgaben des Navigators auf die ambulante Versorgungsphase bei Unterstützungsbedarf in der neuen krankheitsbezogenen Lebenssituation.<br>Die Untersuchung der Machbarkeit dieses Modell soll Erkenntnisse liefern, ob und wie ein derartiges Versorgungsmodell im realen Versorgungssetting implementiert werden kann. Ist die Patientennavigation im realen Versorgungssetting praktikabel und werden Patient*innen mit besonderem Bedarf an Unterstützung über die geplanten Zugangswege und zur passenden Zeit erreicht? Erste Schätzungen zur Wirksamkeit und Kosteneffektivität sollen ergänzend wichtige Erkenntnisse für eine mögliche zukünftige Implementierung von Patientennavigator*innen/-lotsen in der Regelversorgung leisten.
4. Welche der folgenden Bestimmungen finden Anwendung<br>a) Medizinproduktegesetz gemäß § 23b MPG - Ausnahme der klin. Prüfung<br>b) Strahlenschutzgesetz und Strahlenschutzverordnung<br>c) Gendiagnostikgesetz<br>d) Datenschutzgesetze:<br>- Konkrete Angabe des durch die verantwortliche Stelle zu erfüllenden Datenschutzgesetzes (für die Charité =<br>-EU-Datenschutzgrundverordnung (DSGVO), Berliner Datenschutzgesetz - BlnDSG).<br>- Ggf. entsprechend des Teilnehmerkreises zusätzlich zu beachtende Landesdaten-schutzgesetze oder BDSG.	EU-Datenschutzgrundverordnung (DSGVO),<br>Berliner Datenschutzgesetz – BlnDSG<br>Brandenburgisches Datenschutzgesetz – BbgDSG
5. Ggf.: Bezeichnung und Charakterisierung der Prüfprodukte	Patientenorientiertes Navigationsprogramm für Lungenkrebs- und Schlaganfallbetroffene
6. wesentliche Ergebnisse der vorklinischen Tests oder Gründe für die Nichtdurchführung derselben	Entfällt. Keine Untersuchung nach AMG

7. Wesentlicher Inhalt und Ergebnisse der vorangegangenen Studien/Anwendungen der in der Studie zu prüfenden Produkte	Im Rahmen einer ersten Förderphase wurden für die Entwicklung des patientenorientierten Navigationsprogramms Untersuchungen zu Hürden im Versorgungsverlauf aus der Perspektive von Patient*innen und Versorgungsexpert*innen untersucht, bestehende Unterstützungsressourcen in der Region Berlin und Brandenburg zusammengestellt und Untersuchung zu vulnerablen Patient*innen nicht-optimaler Versorgung durchgeführt.<br>Hauptergebnisse dieser Untersuchungen waren:<br>• Versorgungsprozesse sind an institutionelle Bedarfe sowie entlang bürokratischer Strukturen von Versorgungsinstitutionen (Krankenkasse, Krankenhäuser, ambulante Versorgung) angepasst. Dies führt zu hoher Belastung der Patient*innen bei der Versorgungsorganisation<br>• Patient*innen ohne soziale Unterstützungen sind besonders vulnerabel.<br>• Unterstützungsangebote sind in der Region Berlin und Brandenburg für viele Bedarfe vorhanden (siehe hierzu auch die Broschüren mit Unterstützungsangeboten unter https://navicare.berlin/de/ressourcen-fuer-patienten/). Die Kenntnis über die Existenz dieser Angebote ist aber bei Patient*innen und teilweise auch Versorger*innen nicht vorhanden bzw. mit einem hohen Aufwand verbunden.<br>• Patient*innen erfahren Barrieren in der Organisation ihrer Versorgung häufig im ambulanten Bereich.<br>• Versorgung von Lungenkrebspatient*innen im stationären Setting läuft zum hohen Maße entsprechend der Leitlinien ab.
8. Beschreibung der vorgesehenen Maßnahmen/Untersuchungsmethoden und eventuelle Abweichungen von den in der med. Praxis üblichen Maßnahmen/Untersuchungen (was ist „Routine“, was wird davon abweichend in der Studie gemacht?)<br><br>Kommen validierte Fragebögen studienbedingt zum Einsatz, geben Sie bitte die Bezeichnung der Fragebögen an und wo diese publiziert sind (Referenzen).<br>Nicht validierte Fragebögen bitte als Anlage beifügen.	Die Machbarkeit und Wirksamkeit einer patienten-orientierten Patientennavigationsintervention wird im Rahmen einer **Mixed-Methods-Studie im Design einer zweiarmigen, kontrollierten, randomisierten Studie mit parallellaufender Kohortenstudie** untersucht. Erhebungen finden durch **quantitative Befragungen und Datenerfassungen, qualitative Untersuchungen sowie Sekundärdatenanalysen von GKV-Routinedaten** statt. (siehe Abb.2)**.**<br><br>Rekrutierung:<br>Die Studienteilnahme ist für Patient*innen und unter bestimmten Bedingungen auch für Angehörige möglich (als Angehörige gelten Personen aus der Familie sowie weitere dem Patient*innen nahestehende Personen wie Freund*innen oder Nachbar*innen, welche mit der Organisation der Versorgung betraut sind). Angehörige können an der Studie teilnehmen, wenn sie gesetzliche Betreuer*innen einer/eines Patient*in sind.<br>Die Studienteilnehmenden werden in den Rekrutierungszentren (Stroke Units CCM/CVK (und Intensivstation – Amendement aus August 2021), Asklepios Klinikum Brandenburg (Amendement aus August 2021: Geändert zu Klinik Hennigsdorf aufgrund des Ortwechsels des kooperierenden Arztes), Lungentumorambulanz CVK und dem

	Städtischen Klinikum Brandenburg) durch das Studienpersonal angesprochen, aufgeklärt und eingeschlossen. Zudem wird auf den Stationen, die Schlaganfall und – Lungenkrebspatient*innen versorgen, Informationsmaterial ausgelegt werden. Damit haben Interessierte die Möglichkeit auch selbst aktiv den Kontakt zur Teilnahme an der Studie aufzunehmen.<br>Teilnahme aufklärungsfähiger Patient*innen:<br>Patient*in wird für die Studienteilnahme direkt angesprochen. Bei Einschluss erklärt er/sie seine Einwilligung zur Erhebung der medizinischen Basisdaten, der Fragebogenerhebungen, der Erhebung seiner Krankenkassendaten und ggf. qualitativer Daten.<br>Als weitere Möglichkeit kann der/die Angehörige mit dem/der Patient*in als zusätzlicher Studienteilnehmende in die Studie eingeschlossen werden. Hier erklärt der/die Patient*in seine/ihre Einwilligung zur Erhebung seiner/ihrer medizinischen Basisdaten und seiner/ihrer Krankenkassendaten sowie die Einwilligung, dass der/die Angehörige in seinem/ihrem Interesse die Navigation nutzen kann. Der/die Angehörige erklärt seine Einwilligung zur Beantwortung der Fragebogenerhebungen und ggf. qualitativer Daten. Patient*in und Angehörige*r können die Navigation nutzen.<br>Teilnahme nicht aufklärungsfähiger Patient*innen:<br>Ist der/die Patient*in selbst nicht aufklärungsfähig und besteht eine gesetzliche Vertretung durch den/die Angehörige*n, besteht die Möglichkeit der Studienteilnahme durch den/die Angehörige*n. In diesem Fall erteilt der/die Angehörige die Einwilligung für den Patient*innen zur Erhebung seiner/ihrer medizinischen Basisdaten und seiner/ihrer Krankenkassendaten. Für sich selbst erklärt der/die Angehörige seine Erlaubnis zur Beantwortung der Fragebogenerhebungen und ggf. qualitativer Daten. Teilnehmende werden nach Studieneinwilligung durch Randomisierung dem Interventionsarm oder dem Kontrollarm zugeteilt.<br><br>Randomisierung<br>Die Randomisierung erfolgt innerhalb jeder Patientengruppe (Schlaganfall / Lungenkrebs) stratifiziert nach Zentren (Berlin, Brandenburg) und nach Einschluss von Patient*in oder Angehörigem/r als Blockrandomisierung mit wechselnder Blocklänge im Verhältnis 1:1. Nach Einschluss des/der Patient*in durch die Study Nurse, erfolgt die Randomisierung durch das Institut für Biometrie und klinische Epidemiologie der Charité. Die Randomisierungsliste wird von den beteiligten Biometriker*innen erstellt. Nach erfolgter Randomisierung wird die Gruppenzugehörigkeit an die jeweiligen Study Nurses rückgemeldet.<br><br>Vergleichsgruppen:

	• <u>Interventionsgruppe:</u> Erhält die unten beschriebene Navigationsintervention über 1 Jahr ab Erstgespräch mit Navigator. Nach Randomisierung wird die Teilnehmende über die Gruppenzugehörigkeit informiert und erhält Kontaktinformationen des/der Navigator*in.<br>• <u>Kontrollgruppe:</u> Erhält keine Betreuung durch den/die Navigator*in. Nach Randomisierung wird der/die Teilnehmende über die Gruppenzugehörigkeit informiert und erhält die Broschüre mit Unterstützungsangeboten für Schlaganfall- bzw. Lungenkrebsbetroffene und ihre Angehörigen (siehe pdf-Version hier: https://navicare.berlin/de/ressourcen-fuer-patienten/).<br>Patient*innen beider Gruppen können alle üblichen Regelversorgungsleistungen in Anspruch nehmen.<br><br>Patient*innen, welche die Randomisierung im Rahmen des RCT ablehnen, haben die Möglichkeit an einer parallel laufenden Kohortenbefragung teilzunehmen.<br><br>Patient*innen, die die Einschlusskriterien erfüllen, aber an keinem der Studienarme teilnehmen möchten, werden gefragt, ob Sie bereit wären, Ihre Gründe dafür anzugeben. Sie können dann einen „*Refuser*-Fragebogen“ ausfüllen oder ihre Gründe der Study Nurse nennen, die den Fragebogen dann ausfüllt. *Refuser*-Fragebögen können anonym in einer Box auf den Stationen eingeworfen werden oder der Study Nurse gegeben werden. Sie werden vollständig anonym ausgewertet.<br><br><br>Des Weiteren wird die Gesamtzahl der Patient*innen pro Besuch an einem Rekrutierungsstandort sowie Basisdaten (Alter, Geschlecht, ICD-10 Code, Schweregrad/Tumorstadium, Komorbiditäten) über das Krankenhausdokumentationssystem anonym für alle Patient*innen erfasst, um teilnehmende und nicht teilnehmende Patient*innen anhand dieser Parameter vergleichen zu können.<br><br>Anhand von teilnehmender Beobachtung des Rekrutierungsprozesses sowie Experteninterviews mit Study Nurses durch eine wissenschaftliche Mitarbeiterin des Projekts wird der Rekrutierungsprozess flankiert, um mögliche Barrieren, die durch die ausgewählten Settings (Rekrutierungszentren) für die Aufnahme in ein Navigationsmodell entstehen, zu identifizieren.<br><br><u>Intervention</u>:<br>**„Patientenorientiertes Navigationsprogramm für Schlaganfall-/Lungenkrebspatient*innen“:**

	Der/die Navigator*in (Lotse) hat einen beruflichen Hintergrund in der Sozialarbeit (alternativ Pflege, Case-Management, oder eine vergleichbare Ausbildung) und wird entsprechend seiner/ihrer Vorerfahrungen in Bereichen patientenorientierte Versorgung, Kommunikation, bestehendes Unterstützungsangebot für Patient*innen und krankheitsspezifische Aspekte der untersuchten Krankheiten Lungenkrebs und Schlaganfall weiter geschult. Die Schulung ist modular aufgebaut und wird an zwei aufeinanderfolgenden Tagen durchgeführt. Verantwortlich für die Schulung ist das Institut für Sozialmedizin und Epidemiologie der Medizinischen Hochschule Brandenburg. Gemeinsam mit professionellen Partner*innen aus der Versorgung (Sozialarbeiter*innen, Psychoonkolog*innen, Mediziner*innen, Kommunikationswissenschaftler*innen) werden die Navigator*innen in Kommunikation, bestehende Angebote, Barrieren einer patientenorientierten Versorgung, sowie in ihre vernetzende Tätigkeit eingeführt.<br>Für die Navigation nimmt der/die Navigator*in Kontakt mit den rekrutierten Studienteilnehmenden des Interventionsarms auf (bis zu 4 Wochen nach Einschluss) oder wird bei vorherigem Bedarf bereits aktiv durch die Teilnehmenden kontaktiert. Der/die Navigator*in führt ein initiales und persönliches Gespräch mit den Studienteilnehmenden durch, um die individuelle Situation der Studienteilnehmer*in und mögliche bereits bestehende Unterstützungsbedarfe der Teilnehmenden zu identifizieren sowie die Präferenzen der Studienteilnehmenden zur weiteren Form der Interaktion und Kommunikation festzulegen. Der Ort des Gesprächs wird nach Bedürfnissen des/der Studienteilnehmenden festgelegt. Dieses erste Treffen findet standardmäßig in persönlicher Form statt (Amendement aus August 2021: Möglichkeit der Video Konsultation eigeführt aufgrund der Pandemiesituation) und ist von ausreichender Dauer geplant, um die Bedarfe der Studienteilnehmenden identifizieren zu können und einen individuellen Plan der Kommunikation mit den Studienteilnehmenden sowie die initiale Frequenz der Kontakte zu verabreden. Davon unabhängig bieten die Navigator*innen regelmäßige Telefonsprechstunden an, in welchen sie von Studienteilnehmenden kontaktiert werden können. Die Navigation ist für die Dauer von 1 Jahr pro Studienteilnehmenden geplant und die minimale Kontaktfrequenz zwischen Navigator*in und Studienteilnehmenden ist einmal alle 3 Monate.<br><br>Aufgaben der Navigation:<br>(a) Bereitstellung von Informationen zur Unterstützung des Alltags und der weiteren Behandlungsmöglichkeiten, wie Beratungsangebote, psychosoziale und sonstige Unterstützungsmöglichkeiten<br>(b) Hilfe bei Antragsformalien, wie Beantragung von Behindertenausweis oder Pflegegrad und Rehabilitationsmaßnahmen

	(c) Wenn notwendig, Kontaktherstellung zu (Fach)Ärzt*innen, Therapeut*innen und anderen Unterstützungs- und Versorgungseinrichtungen<br><br>Der/die Navigator*in nimmt eine ausführliche Dokumentation des Navigationsprozesses mit den Studienteilnehmenden vor, um Daten für ausgewählten Outcomes zur Untersuchung der Machbarkeit des Navigationsmodells zu erhalten.<br><br>Die Navigator*innen haben wöchentliche Teamsitzungen zur gegenseitigen Unterstützung und zum Informationsaustausch. Diese Sitzungen werden durch teilnehmende Beobachtung einer wissenschaftlichen Mitarbeiterin begleitet. Darüber hinaus werden Experteninterviews mit den Navigator*innen durchgeführt sowie auch leitfaden-gestützte Interviews mit Studienteilnehmenden, um zu erfassen, wie die Navigator*innen Studienteilnehmende unterstützen und wie dies von Studienteilnehmenden und Navigator*innen erlebt wird.<br>Dies dient der Prozessevaluation sowie der Identifikation möglicher Änderungsnotwendigkeiten des Navigationsmodells.<br><br><u>Weitere Datenerhebungsmethoden</u><br>a. <u>Datenerhebung RCT</u><br><u>Fragebogen Assessments:</u><br>Zur Untersuchung der Wirksamkeit der Intervention erhalten beide Gruppen ein Baseline-Assessment am Tag des Studieneinschlusses (sollte dies in Einzelfällen nicht umsetzbar sein, kann dies im Zeitraum bis zu 4 Wochen nach Studieneinschluss durchgeführt werden). Follow-up Befragungen finden 4 Monate, 7 Monate und 13 Monate nach Einschluss statt. (Amendement aus August 2021 und Januar 2022: Vitalstatus der Patient:innen wird beim Einwohnermeldeamt abgefragt vor den Follow-Ups und nach Studienende um Lost-to-Follow-Up Teilnehmer zu identifizieren)<br><br>Die Fragebögen können schriftlich durch den Studienteilnehmenden oder bei Bedarf mit Hilfe von Angehörigen oder des Studienpersonals beantwortet werden.<br><br><u>Sekundärdatenanalyse</u><br>Die AOK Nordost stellt für alle Teilnehmer*innen des RCT, welche bei der AOK Nordost versichert sind, pseudonymisierte Routinedaten zur Verfügung. Diese Daten enthalten Informationen zu individuellen Behandlungs- und Pflegedaten, der Inanspruchnahmehäufigkeit von Leistungen des Gesundheitswesens sowie Daten zu den hiermit assoziierten Kosten. Die Daten umfassen den Zeitraum der Intervention sowie des vorangegangenen Jahres (insgesamt 25 Monate). Der Gesamtdatensatz wird frühestens sechs Monate

	nach Abschluss der Intervention zur Verfügung gestellt (last patient out). Ein Teildatensatz wird etwa sechs Monate nach dem letzten Einschluss der Teilnehmer*innen zur Verfügung gestellt.<br><br>b. <u>Datenerhebung Kohortenstudie</u><br>Die Assessmentzeitpunkte und -inhalte der Befragungen in der Kohortenstudie verlaufen parallel zu den Assessments des RCT mit: Baseline-Befragung bei Einschluss, Follow-Up Befragungen nach 4 Monaten, 7 Monaten und 13 Monaten.<br>Zudem werden leitfaden-gestützte Interviews mit Teilnehmenden der Kohortenstudie durchgeführt, um zu erfahren, wie diese sich im Versorgungsalltag zurechtfinden.
9. Bewertung und Abwägung der vorhersehbaren Risiken und Nachteile der Studienteilnahme gegenüber dem erwarteten Nutzen für die Studienteilnehmer und zukünftig erkrankte Personen (Nutzen-Risiko-Abwägung)	**RCT**<br>Die derzeit zu erwartenden Risiken und Nachteile der Teilnahme am RCT sind:<br>- Gefühl der Überforderung durch die Interaktion mit Navigator*in und Studienpersonal während der Assessmentbefragungen in einer emotional belastenden Krankheitsphase.<br>- Gefühl der Enttäuschung bei Randomisierung in Kontrollarm der Studie ohne Betreuung durch Navigator*in (keine Verblindung möglich)<br>- Zeitlicher Aufwand durch Befragungen zu Baseline und Follow-Up Zeitpunkten von max. 1 Stunde<br>Diesen möglichen Risiken steht der vermutete Nutzen des Navigationsprogramms für die Teilnehmenden im Interventionsarm gegenüber. Durch die Navigation können Unsicherheiten und Barrieren im Versorgungsverlauf der Patient*innen kurzfristig abgebaut werden. Auch Teilnehmende in der Kontrollgruppe erhalten Unterstützung in Form der Broschüre mit Unterstützungsangeboten für Schlaganfall-/Lungenkrebspatient*innen, die eine Orientierung geben kann.<br>Langfristiger Nutzen ist der Erkenntnisgewinn zur Machbarkeit und Wirksamkeit des Patientennavigationsmodells, welcher langfristig zur Implementierung von Navigations-/Lotsenangeboten in die Regelversorgung und somit zum Abbau von Hürden in der Versorgungskoordination in der deutschen Versorgungslandschaft beitragen kann.<br><br>**Kohortenbefragung**<br>Die Befragungen zur Baseline und den Follow-Up Zeitpunkten nehmen max. 1 Stunde pro Befragungszeitpunkt in Anspruch. Dem gegenüber steht der langfristige Erkenntnisgewinn zur Machbarkeit und Bedarf an Navigation bei den untersuchten Patientenkohorten. Dies rechtfertigt den zeitlichen Aufwand der Befragungen.<br><br>**Interviews mit Studienteilnehmer*innen:**<br>Der zeitliche Rahmen für die Interviews mit den Studienteilnehmer*innen ist abhängig von der

	Erzählweise und dem Erzählbedürfnis. In der Regel dauern solche Interviews ca. 60 Minuten. Die Interviews können dazu führen, dass sich die Studienteilnehmer*innen an unangenehme Situationen erinnern oder auch in die Auseinandersetzung mit ihrer Erkrankung und ihrer Versorgung kommen. Dies kann sowohl positive als auch negative Konsequenzen für sie haben. Viele Studienteilnehmer*innen berichten, dass es für sie eine Erleichterung war, „einmal erzählen zu dürfen" und so auch Sinnzusammenhänge in dem Geschehenen zu erfahren (Literatur: Dvorak F. Krankheitserfahrungen erzählen. Evaluation der Teilnahme an einer patientenorientierten Gesundheitswebseite aus der Perspektive der Interviewten. Freiburg: Institut für Psychologie; 2010). Demgegenüber steht die Möglichkeit, dass Studienteilnehmer*innen erst durch das Interview negativer Erlebnisse und Emotionen sowie Einschränkungen gewahr werden. Die Interviews werden von in Interviewführung geschulten und sehr erfahrenen wissenschaftlichen Mitarbeiter*innen durchgeführt. Diese ziehen bei Bedarf die Psychoonkologie des CCCC zur Supervision hinzu bzw. vermitteln die Interviewpartner*innen an die Psychoonkologie des CCCC. Häufig tritt auch im Nachgang des Interviews noch Bedarf auf. Hierfür werden den Studienteilnehmer*innen Kontaktdaten für psychologische Unterstützung gegeben.<br><br>**Experteninterviews mit Study Nurses und Navigator*innen:**<br>Mit den Befragungen der Study Nurses zu ihren Erfahrungen zum Rekrutierungsprozess ist ein zeitlicher Aufwand verbunden (ca. 30-45 Min. pro Interview). Die Befragungen können unter Umständen zu Gefühlen wie Frustration und Ärger führen, wenn sie eher negative Erlebnisse damit verbinden. Andererseits können das Erzählen und die Reflexion darüber auch hilfreich sein, solche Erfahrungen besser verarbeiten zu können. Für die Implementierung des Navigationsmodells ist es von entscheidender Bedeutung herauszufinden, ob die gewählten Settings zur Rekrutierung (und damit für die Aufnahme von Teilnehmenden in das Navigationsprogramm) geeignet sind. Daher ist der zeitliche Aufwand für die Interviews gerechtfertigt. Die Study Nurses werden bei Bedarf professionell durch die Psychoonkologie des CCCC begleitet, um ggf. psychologische Unterstützung bieten zu können.<br><br>Mit den Befragungen der Navigator*innen ist ein zeitlicher Aufwand verbunden (ca. 30-45 Min. pro Interview). Die Befragungen können bei den Navigator*innen zu emotionalen Belastungen führen, wenn sie sich beispielsweise an unangenehme Situationen erinnern und Gefühle wie Hilflosigkeit (z.B. wenn die Navigator*innen die Erfahrung machen sollten, Studienteilnehmer*innen nicht helfen zu können), Überforderung oder Frustration in ihnen hervorgerufen werden. Neben der wöchentlichen Team-Sitzung, die als Peer-Support eingerichtet ist,

	besteht auch hier die Möglichkeit bei Bedarf professionell durch die Psychoonkologie des CCCC Unterstützung zu erhalten. Die Befragungen werden häufig als hilfreich erlebt, da sie die Selbstreflexion stärken und bestehende Probleme/Unzufriedenheiten aufzulösen. Die Gespräche und Interviewprotokolle werden nur in ausgewerteter Form mit der Projektleitung geteilt. Die Projektleitung hat keinen Zugriff auf die Originaldaten der Navigator*innen, des Rekrutierungsprozesses, der Team-Sitzungen oder der Interviews mit Study Nurses. Nur die Ergebnisse werden mit der Projektleitung besprochen.<br>Die Befragung der Navigator*innen zur Durchführbarkeit/Machbarkeit des Navigationsprogramms sowie zu ihren Erfahrungen als Erbringer*innen der Navigationsleistungen ist ein zentraler Baustein der Evaluation der Intervention und rechtfertigt daher den Aufwand und die möglichen Risiken.<br><br>**Teilnehmende Beobachtung:**<br>Durch die teilnehmende Beobachtung einer wissenschaftlichen Mitarbeiterin des Projekts während der Rekrutierungsphase soll evaluiert werden, ob die gewählten Settings für die Aufnahme von Teilnehmer*innen in ein Navigationsprogramm geeignet sind und wie das Navigationsprogramm dargestellt und angenommen wird. Unter Umständen fühlen sich die Study Nurses nicht ganz so frei in ihrer Arbeit wie ohne Beobachtung, wobei aber dieses Risiko versucht wird zu minimieren, indem die teilnehmende Beobachtung durch eine zum Team gehörige Projektmitarbeiterin (und nicht durch eine fremde Person) durchgeführt wird.<br>Zudem werden die regelmäßig stattfindenden Teammeetings der Navigator*innen durch teilnehmende Beobachtung einer wissenschaftlichen Mitarbeiterin des Projekts begleitet. Unter Umständen könnten sich die Navigator*innen etwas gehemmt fühlen durch die Anwesenheit der beobachtenden Person, allerdings handelt es sich um eine zum Projekt gehörige wissenschaftliche Mitarbeiterin, die den Navigator*innen bekannt ist, was dieses Risiko minimieren sollte.<br>Durch die teilnehmende Beobachtung des Rekrutierungsprozesses sowie der Teamsitzungen der Navigator*innen sollen wichtige Rückschlüsse in Bezug auf die Durchführung und Machbarkeit des Navigationsprogramms gezogen werden. Dies rechtfertigt den Aufwand und die Risiken.
a. zu prüfender medizinischer Nutzen für die Studienteilnehmer (individueller Nutzen für den einzelnen Patienten)	Nutzen durch die Patientennavigation in Bezug auf die Verbesserung der gesundheitsbezogenen Lebensqualität oder die Zufriedenheit mit der Versorgung werden im Rahmen der Studie untersucht.<br>Zudem haben bisherige Studien aus dem internationalen Kontext gezeigt, dass Patientennavigationsmodelle auch positive Effekte auf die Versorgungskoordination sowie die Häufigkeit der

	Hospitalisierungen der Patient*innen haben können, was im Rahmen der Sekundärdatenanalysen mit Krankenkassendaten betrachtet wird.<br>Teilnehmende des Interventionsarms können also durch den/die Navigator*in profitieren, welche*r beim Abbau von Hürden in der persönlichen Versorgungskoordination und- organisation unterstützen soll und so zu einer besseren Einsteuerung der komplexen Nachversorgung beiträgt. Dies kann zu Abbau von Ängsten, Stress, schnellerem und zielgerichteten Zugang zu Versorgungsleistungen führen. Auch die Teilnehmenden der Kontrollgruppe erhalten mit der Broschüre mit Unterstützungsangeboten eine Hilfestellung, welche zur besseren Kenntnis des Unterstützungsangebots und somit zum Abbau von Fragen und Hürden bezüglich der Erkrankung führen kann.<br>Im Rahmen der Interviews mit Studienteilnehmenden kann das Sprechen über eigene Erfahrungen für Studienteilnehmer*innen hilfreich sein, das Erlebte zu verarbeiten. Auch für Study Nurses und Navigator*innen kann das Sprechen über die erlebten Erfahrungen im Zusammenhang mit der eigenen Arbeit im Rahmen des Navigationsprogramms der Selbstreflexion dienen und damit hilfreich sein. Auch die Rückspiegelung der Beobachtungen der teilnehmenden Beobachtung kann für Study Nurses und Navigator*innen hilfreich sein, die eigenen Erfahrungen und Erlebnisse zu reflektieren, zu verarbeiten und Verbesserungen für die eigene Arbeit anstoßen.
b. zu prüfender medizinischer Nutzen für zukünftig erkrankte Personen (Gruppennutzen)	Ziel der Studie ist es, die Machbarkeit (und Wirksamkeit) eines patientenorientierten Patientennavigationsmodells für Patient*innen mit altersassoziierten Erkrankungen (am Beispiel der beiden prototypischen Erkrankungen Schlaganfall und Lungenkrebs) zu untersuchen. Bei erfolgreicher Machbarkeit kann die Studie wichtige Erkenntnisse für die Implementierung einer solchen Navigations-/Lotsenintervention im deutschen Versorgungsumfeld liefern. Dies kann langfristig zur Verbesserung der Organisation von Versorgungsabläufen und als sekundärer Effekt auch zu einer generell besseren Vernetzung und Bekanntheit der regionalen Versorgungs- und Unterstützungsangebote führen, wodurch perspektivisch eine verbesserte Nutzung dieser Angebote bei bestehendem Bedarf durch Patient*innen und Angehörige erreicht werden könnte.
c. **Risiken** und Belastungen für die Studienteilnehmer (alle im Einzelnen auflisten)	**RCT**<br>Die Teilnahme am RCT der Studie kann zu Gefühlen der Überforderung durch mehrfache Follow-Up Befragungen führen.<br>Zudem kann es zu Gefühlen der Enttäuschung und des Alleingelassenseins führen, wenn der/die Studienteilnehmer*in in die Kontrollgruppe ohne Betreuung durch den/die Navigator*in randomisiert wird.

	**Kohorte**<br>Die Teilnahme am RCT der Studie kann zu Gefühlen der Überforderung durch mehrfache Follow-Up Befragungen führen.<br><br>**Interviews mit Studienteilnehmer*innen:**<br>Die Interviews können dazu führen, dass sich Studienteilnehmer*innen an unangenehme Situationen erinnern oder auch mit der Auseinandersetzung mit ihrer Erkrankung und ihrer Versorgung konfrontiert werden.<br><br>**Sekundärdatenanalyse**<br>Die retrospektive Analyse der pseudonymisierten Kassendaten der AOK-versicherten Teilnehmer*innen stellt für diese kein Risiko oder Belastung dar.
10. Maßnahmen zur Risikobeherrschung	Die Teilnahme an allen Studienteilen ist freiwillig. Die Teilnehmenden werden darauf hingewiesen, dass sie die Studienteilnahme jederzeit ohne Angabe von Gründen abbrechen können. Alle erhobenen Daten werden in pseudonymisierter Form ausgewertet und berichtet.<br><br>Das Studienpersonal und die Navigator*innen werden professionell geschult, Belastungen auf Seiten der Studienteilnehmenden erkennen zu können.<br>Auch im Nachgang der Navigationsintervention/Studienteilnahme kann Unterstützungsbedarf entstehen. Daher werden den Patient*innen und ihren Angehörigen Kontaktdaten für Unterstützung gegeben. Diese Information ist in der Studieninformation verfügbar.<br><br>Für die Lungenkrebspatient*innen:<br>- Hotline des CCCC:<br>- Telefonnummer der Leiterin der Psychoonkologie am CCCC [aufgrund von Datenschutzgründen gelöscht für PLOS One Protocoll Publikation]<br><br>Für die Schlaganfallpatient*innen:<br>- Beratungstelefon des Servicepunkt Schlaganfall: [aufgrund von Datenschutzgründen gelöscht für PLOS One Protocoll Publikation]<br><br>Zudem sind diese und weitere Kontaktadressen in der Broschüre mit Unterstützungsangeboten angegeben.
11. Abbruchkriterien	• Teilnehmende*r zieht Einwilligung zurück<br>• Studienpersonal bemerkt eine psychische Belastung, die einen Abbruch notwendig macht. Dies geschieht dann in Absprache mit der Psychoonkologie des CCCC bzw. den ärztlichen Ansprechpartner*innen der Neurologie.
12. Anzahl, Alter und Geschlecht der betroffenen Personen	Geplante Fallzahlen:<br>1. Schlaganfall<br>Insgesamt sollen 685 Schlaganfallpatient*innen und ggf. Angehörige (männlich/weiblich/divers, Alter: ≥18 Jahre) in die Gesamtstudie eingeschlossen werden. Diese teilen sich wie folgt auf die Studienteile auf:

	a. RCT<br>460 Schlaganfallpatient*innen (und ggf. Angehörige)<br><br>b. Kohortenstudie<br>225 Schlaganfallpatient*innen,<br><br>c. Qualitative Studie<br>Studienteilnehmende aus RCT und Kohortenstudie (zudem jeweils 3 Study Nurses/Navigatoren (m/w/d) ab 18 Jahre)<br><br>d. Sekundärdatenanalyse<br>Ca. 165 Schlaganfallpatient*innen aus RCT (abhängig vom tatsächlichen AOK-Versichertenanteil im RCT),<br><br>2. Lungenkrebs<br>Insgesamt sollen 195 Lungenkrebspatient*innen und ggf. Angehörige (männlich/weiblich/divers, Alter: ≥18 Jahre) in die Gesamtstudie eingeschlossen werden. Diese teilen sich wie folgt auf die Studienteile auf:<br>a. RCT<br>120 Lungenkrebspatient*innen (und ggf. Angehörige)<br><br>b. Kohortenstudie<br>75 Lungenkrebspatient*innen<br><br>c. Qualitative Studie<br>Studienteilnehmende aus RCT und Kohortenstudie (zudem jeweils 3 Study Nurses/Navigatoren (m/w/d) ab 18 Jahre)<br><br>d. Sekundärdatenanalyse<br>Ca. 43 Lungenkrebspatient*innen aus RCT (abhängig vom tatsächlichen AOK-Versichertenanteil im RCT)
13. Biometrische Planung mit Angabe der statistischen Methodik, einschließlich der Begründung der Fallzahl.<br>Angabe des/der Statistikers/Statistikerin (sofern Beratung durch das Institut für Biometrie der Charité erfolgt, muss eine Unterschrift eingefügt werden)	Biometrische Planung:<br>Biometrische Berechnungen wurden basierend auf den zwei definierten primären Machbarkeitsoutcomes zur Bestimmung der Machbarkeit berechnet. Diese lauten wie folgt:<br>„Die Intervention ist machbar, wenn:<br>(1) Mindestens 70% der Patient*innen des Interventionsarms an wenigstens einer initialen persönlichen Navigatorsitzung teilgenommen haben.<br>UND<br>(2) Die Abbruchrate des Interventionsarms des RCT weniger als 40% beträgt (hierbei sind Abbrecher definiert als Studienteilnehmende, welche die Intervention aus anderen Gründen abbrechen, als jene die Patient*innen/Angehörigen physisch von der Teilnahme abhalten. Z.B. Umzug aus

	dem Einzugsgebiet, Verschlechterung des allgemeinen Gesundheitszustands, Langzeit-Hospitalisierung, Umzug in Pflegeheim oder Hospiz, Tod).<br><br>Erläuterungen in den einzelnen Studienteilen:<br>1. Schlaganfall<br>a. RCT<br>Für die Dauer der Rekrutierungszeit rechnen wir auf Basis von Daten vorheriger Jahre mit einer Grundgesamtheit von etwa 1850 Schlaganfallpatient*innen an den drei geplanten Rekrutierungsstandorten (1100 Patient*innen an Charité Universitätsmedizin Berlin Stroke Units an den Standorten Mitte und Virchow Klinikum, 750 Patient*innen an Asklepios Fachklinikum Brandenburg). Wir gehen davon aus, dass von diesen Patient*innen 70% durch das Studienpersonal für die Teilnahme an der Studie angesprochen werden können. Von den angesprochenen Patient*innen rechnen wir mit einer Rekrutierungsrate (je nach Standort) von 30-50%. Basierend auf diesen Vorannahmen rechnen wir mit einer Teilnahme von 460 Schlaganfallpatient*innen (und ihren Angehörigen).<br>Bezogen auf die oben definierten Machbarkeitskriterien ergibt sich folgende statistische Planung:<br>Wenn 460 Schlaganfallpatient*innen in die Studie eingeschlossen werden, und 230 (50%) der Patient*innen in den Interventionsarm randomisiert werden, gehen wir davon aus, dass mindestens 95% (n=219) nach 4 Wochen noch für den Erhalt der Patientennavigation überlebt haben. Wenn 166 (75,8%) oder mehr dieser 219 Patient*innen die initiale Navigationssitzung erhalten, ist das erste Machbarkeitskriterium eingehalten, da das 95% Konfidenzintervall dieses Anteils nicht unter 70% sein wird (95%KI: 70,1%-81,5%). Wir nehmen zusätzlich an, dass von diesen 230 Patient*innen (Interventionsarm), 85% (n=196) nach einem Jahr überlebt haben. Wenn von diesen 196 Patient*innen, 65 (33,2%) oder weniger „lost-to-follow-up“ sind, wird das zweite Machbarkeitskriterium erreicht, da das 95% Konfidenzintervall dieses Anteils kleiner als 40% sein wird (95% KI: 26,6%-39,8%).<br>Die Machbarkeit der Studie wird als erfolgreich angesehen, wenn beide Kriterien erreicht werden.<br><br>Im Falle der erfolgreich nachgewiesenen Machbarkeit, testen wir zusätzlich in der Studie zum zweiseitigen Signifikanzniveau α=0.05 die Efficacy der Intervention mittels ANCOVA, adjustiert für Baseline Messung eines ausgewählten patientenberichteten Outcomes (z.B. gesundheitsbezogene Lebensqualität) (Anm. d. Autors: Hier wurde als Outcome ‘Zufriedenheit mit der Versorung’ im Rahmen der Studienregistrierung vor

	Rekrutierungsstart definiert) und adjustiert für die Stratifizierungsvariablen der Randomisierung (hierarchisches Testen). Alle primären Fragestellungen werden konfirmatorisch im „full analysis set“ analysiert. Im Falle von fehlenden Werten und bei Annahme von „missing at random“ werden multiple Imputationsmodelle verwendet, um die fehlenden Werte zu schätzen.<br><br>Neben den eingangs beschriebenen Analysen der primären Outcomes, werden weitere Machbarkeitskriterien als sekundäre Outcomes deskriptiv/explorativ ausgewertet und patientenberichtete Outcomes zwischen den Interventionsarmen in univariaten Analysen und multiplen Regressionsmodellen verglichen (siehe Tabellen 1-3). Explorative Subgruppenanalysen werden durchgeführt, um Unterschiede in der Machbarkeit und Effektivität in Bezug auf prädefinierte Komparatoren zu untersuchen.<br><br>b. Kohortenstudie<br>Wir erwarten bei der Kohortenstudie eine Teilnahmerate von 30% der angesprochenen Patient*innen, welche eine Teilnahme im RCT ablehnen. Hieraus ergibt sich eine Fallzahl von 225 Schlaganfallpatient*innen in der Kohortenstudie.<br><br>Erhobene Daten der patientenberichteten Outcomes werden explorativ/deskriptiv ausgewertet und longitudinal verglichen.<br><br>c. Prozessevaluation<br>Für die Prozessevaluation inklusive der qualitativen Interviews mit Studienteilnehmer*innen ist keine biometrische Planung notwendig, vielmehr wird zur Abbildung der Erfahrungen mit der Intervention ein Sampling vorgenommen, bis die Studienergebnisse als saturiert gelten. Erfahrungen aus anderen qualitativen Studien legen nahe, dass mit der Gesamtzahl von 30 – 40 Interviews ein gutes Bild der verschiedenen Erfahrungen mit der Intervention abgebildet werden kann. Auswahlkriterien sind jeweils Alter, Geschlecht, Schweregrad der Erkrankung, Familienstatus (alleinlebend vs. mit Partner*in zusammenlebend), Vorhandensein vs. Nicht-Vorhandensein sozialer Unterstützung, Zufriedenheit vs. Nicht-Zufriedenheit mit Navigation; Schwierigkeiten mit der Navigation.<br><br>d. Sekundärdatenanalyse<br>Ausgehend von den angenommenen 460 in das RCT eingeschlossene Patient*innen und dem durchschnittlichen Anteil von AOK-Versicherten von ca. 36% (Stand 2018) in der Gesamtbevölkerung Deutschlands erwarten wir für die Sekundärdatenanalyse von Kassendaten eine Fallzahl von 165.

	Die Daten werden explorativ/deskriptiv ausgewertet und longitudinal verglichen. Den üblicherweise nicht-normalverteilten Kostendaten wird in den statistischen Analysen mit entsprechenden Verfahren Rechnung getragen.<br><br>2. <u>Lungenkrebs</u><br>a. RCT<br>Für die Dauer der Rekrutierungszeit rechnen wir mit einer Grundgesamtheit von etwa 550 Lungenkrebspatient*innen an den drei geplanten Rekrutierungsstandorten (Charité Universitätsmedizin Lungentumorambulanz am Standort Virchow Klinikum, 50 Patient*innen am Städtischen Klinikum Brandenburg). Wir gehen davon aus, dass von diesen Patient*innen 70% durch das Studienpersonal für die Teilnahme an der Studie angesprochen werden können. Von den angesprochenen Patient*innen rechnen wir mit einer Rekrutierungsrate (je nach Standort) von 30-50%. Basierend auf diesen Vorannahmen rechnen wir mit einer Teilnahme von 120 Lungenkrebspatient*innen (und ihren Angehörigen)<br>Bezogen auf die oben definierten Machbarkeitskriterien ergibt sich folgende statistische Planung:<br>Wenn 120 Lungenkrebspatient*innen in die Studie eingeschlossen werden, und 60 (50%) der Patient*innen in den Interventionsarm randomisiert werden, gehen wir davon aus, dass mindestens 95% (n=57) nach 4 Wochen noch für den Erhalt der Patientennavigation überlebt haben. Wenn 46 (80%) oder mehr dieser 57 Patient*innen die initiale Navigationssitzung erhalten, ist das erste Machbarkeitskriterium eingehalten, da das 95% Konfidenzintervall dieses Anteils nicht unter 70% sein wird (95%KI: 70,5%-90,9%). Wir nehmen zusätzlich an, dass von diesen 57 Patient*innen (Interventionsarm), 75% (n=43) nach einem Jahr überlebt haben. Wenn von diesen 43 Patient*innen, 11 (25,6%) oder weniger „lost-to-follow-up“ sind, wird das zweite Machbarkeitskriterium erreicht, da das 95% Konfidenzintervall dieses Anteils kleiner als 40% sein wird (95% KI: 12,5%-38,6%)<br>Die Machbarkeit der Studie wird als erfolgreich angesehen, wenn beide Kriterien erreicht werden.<br><br>Analog zur Schlaganfallstudie wird im Falle der erfolgreich nachgewiesenen Machbarkeit die Efficacy der Intervention in Bezug ein ausgewähltes patientenberichtetes Outcomes (z.B. gesundheitsbezogene Lebensqualität) (Anm. d. Autors: Hier wurde als Outcome ‘Zufriedenheit mit der Versorung’ im Rahmen der <u>Studienregistrierung</u> vor Rekrutierungsstart definiert) zusätzlich als primäre Fragestellung zum zweiseitigen Signifikanzniveau von

	α =0.05 getestet (hierarchisches Testen). Kann die Machbarkeit nicht nachgewiesen werden, werden alle weiteren Outcomes sekundär explorativ analysiert.<br><br>Neben den eingangs beschriebenen Analysen der primären Outcomes, werden weitere Machbarkeitskriterien als sekundäre Outcomes deskriptiv/explorativ ausgewertet und patienten-berichtete Outcomes zwischen den Interventionsarmen in univariaten Analysen und multiplen Regressionsmodellen verglichen (siehe Tabellen 1-3). Explorative Subgruppenanalysen werden durchgeführt, um Unterschiede in der Machbarkeit und Effektivität in Bezug auf prädefinierte Komparatoren zu untersuchen.<br><br>b. Kohortenstudie<br>Wir erwarten bei der Kohortenstudie eine Teilnahmerate von 30% der angesprochenen Patient*innen, welche eine Teilnahme im RCT ablehnen. Hieraus ergibt sich eine Fallzahl von 75 Lungenkrebspatient*innen in der Kohortenstudie.<br><br>Erhobene Daten der patientenberichteten Outcomes werden explorativ/deskriptiv ausgewertet und longitudinal verglichen.<br><br>c. Prozessevaluation<br>Für die Prozessevaluation inklusive der qualitativen Interviews mit Studienteilnehmer*innen ist keine biometrische Planung notwendig, vielmehr wird zur Abbildung der Erfahrungen mit der Intervention ein Sampling vorgenommen, bis die Studienergebnisse als saturiert gelten. Erfahrungen aus anderen qualitativen Studien legen nahe, dass mit der Gesamtzahl von 30 – 40 Interviews ein gutes Bild der verschiedenen Erfahrungen mit der Intervention abgebildet werden kann. Auswahlkriterien sind jeweils Alter, Geschlecht, Schweregrad der Erkrankung, Familienstatus (alleinlebend vs. mit Partner*in zusammenlebend), Vorhandensein vs. Nicht-Vorhandensein sozialer Unterstützung, Zufriedenheit vs. Nicht-Zufriedenheit mit Navigation; Schwierigkeiten mit der Navigation.<br><br>d. Sekundärdatenanalyse<br>Ausgehend von den angenommenen 120 in das RCT eingeschlossenen Patient*innen und dem durchschnittlichen Anteil von AOK-Versicherten von ca. 36% (Stand 2018) in der Gesamtbevölkerung von Berlin und Brandenburg erwarten wir für die Sekundärdatenanalyse von Kassendaten eine Fallzahl von 43 aus.<br>Die Daten werden explorativ/deskriptiv ausgewertet und longitudinal verglichen. Den üblicherweise nicht-normalverteilten Kostendaten wird in den statistischen

	Analysen mit entsprechenden Verfahren Rechnung getragen.
14.<br>a. Darlegung und ggf. Erläuterung der **Ein- und Ausschlusskriterien**	Ein-/Ausschlusskriterien:<br>1. Schlaganfall<br>a. RCT<br>In die Studie eingeschlossen werden können alle Schlaganfallpatient*innen, welche in den Rekrutierungszentren und im Rekrutierungszeitraum von 1 Jahr behandelt werden, sowie deren Angehörige. Aufgrund der Untersuchung im realen Versorgungssetting und -umfeld soll kein regelhafter Ausschluss von Patient*innen mit Komorbiditäten durchführt werden. Ausnahmefälle hierbei sind in den Ausschlusskriterien aufgeführt.<br>Einschlusskriterien:<br>• Bestätigte Diagnose Schlaganfall/TIA (ICD-10 Codes: G45.x, I60.x, I61.x, I63.x, I64.x) (H34.x (Amendement aus August 2021)), (H47.0 (Amendement aus Mai 2022))<br>• Angehörige*r einer/eines Patient*in mit Schlaganfalldiagnose (bei Einwilligung durch den/die Patient*in oder bestehender gesetzlicher Vertretung)<br>• Alter: ≥18 Jahren<br>• wohnhaft in Berlin und Brandenburg<br><br>Ausschlusskriterien:<br>• Pflegeheimbewohner*in zum Zeitpunkt des Einschlusses<br>• Patient*innen ohne Angehörige, welche nicht aufklärungsfähig sind, und bei denen keine bestehende gesetzliche Betreuung durch den/die Angehörige*n besteht<br>• Demenzerkrankungen (hier aber Einschluss der Angehörigen möglich)<br>• Sprachbarriere (hier aber Einschluss der Angehörigen möglich)<br><br>b. Kohortenstudie<br>Wie a, aber keine Teilnahme durch Angehörige geplant<br><br>c. Qualitative Studie<br>Studienteilnehmende aus a und b.<br><br>d. Sekundärdatenanalyse<br>In diesem Studienteil werden alle RCT-Teilnehmende eingeschlossen, welche bei der AOK Nordost versichert sind.<br>Einschlusskriterien:<br>• Teilnahme am RCT<br>• Versichert bei der AOK Nordost<br><br>2. Lungenkrebs

	a. RCT<br>In die Studie eingeschlossen werden können alle Lungenkrebspatient*innen, welche in den Rekrutierungszentren und im Rekrutierungszeitraum von 1 Jahr behandelt werden sowie deren Angehörige. Aufgrund der Untersuchung im realen Versorgungssetting und -umfeld soll kein regelhafter Ausschluss von Patient*innen mit Komorbiditäten durchführt werden. Ausnahmefälle hierbei sind in den Ausschlusskriterien aufgeführt.<br>Einschlusskriterien:<br>• Bestätigte Diagnose Lungenkrebs (ICD-10 Codes: C34.1, C34.2, C34.3, C34.8, C34.9, C97)<br>• Angehörige*r einer/eines Patient*in mit Lungenkrebsdiagnose (bei Einwilligung durch den/die Patient*in oder bestehender gesetzlicher Vertretung)<br>• Alter: ≥18 Jahren<br>• wohnhaft in Berlin und Brandenburg<br><br>Ausschlusskriterien:<br>• Pflegeheimbewohner*in zum Zeitpunkt des Einschlusses<br>• Patient*in ohne Angehörige, welche nicht aufklärungsfähig sind, und bei denen keine bestehende gesetzliche Betreuung durch den/die Angehörige*n besteht<br>• Demenzerkrankungen (hier aber Einschluss der Angehörigen möglich)<br>• Sprachbarriere (hier aber Einschluss der Angehörigen möglich)<br><br>b. Kohortenstudie<br>Wie a, aber keine Teilnahme durch Angehörige geplant.<br><br>c. Qualitative Studie<br>Studienteilnehmende aus a und b.<br><br>d. Sekundärdatenanalyse<br>In diesem Studienteil werden alle RCT-Teilnehmer*innen eingeschlossen, welche bei der AOK Nordost versichert sind und ihre Zustimmung zur Abfrage der GKV-Routinedaten gegeben haben.
b. **Studieninformation** (wer diese mündlich und schriftlich erteilt und Angabe, wie viel Zeit zwischen Aufklärung und Einwilligung verbleibt (schriftliche Information als Anlage)	Aufklärung wird mündlich durch Studienmitarbeiter*innen erteilt. Zudem erhält der/die Patient*in eine schriftliche Studieninformation.<br>Der/die Patient*in bzw. Angehörige erhält nach Aufklärung ausreichend Zeit zur Klärung von offenen Fragen. Es besteht die Möglichkeit des Studieneinschlusses am Tag der Aufklärung und nach Wunsch auch die Möglichkeit einer späteren Kontaktaufnahme mit dem/durch das Studienteam und Einschluss des/der Patient*in/Angehörigen.

	Hierfür erhält der/die Patient*in einen Informationsflyer zur Studie inklusive Kontaktinformation.<br>Nach Aufklärung hat der/die Studienteilnehmer*in die Wahl zur Teilnahme am RCT-Teil oder am Kohortenteil der Studie.
c. **Einwilligungserklärung** (schriftliche Form als Anlage)	Einwilligungen und Kontaktformular
d. Ggf. **Information und Einwilligung des gesetzlichen Vertreters** (ggf. auch Beschreibung des Verfahrens zur Einrichtung einer gerichtlichen Betreuung)	Falls der/die Patient*in gesetzlich betreut wird, wird die Einwilligung des/der gesetzlichen Vertreter*in eingeholt.
15. Maßnahmen zur Gewinnung von Studienteilnehmern (Aushang?, Zeitungsannoncen? Etc.)	Schlaganfall:<br>Zur Kontaktherstellung und Rekrutierung der Studienteilnehmenden werden die bereits bestehende Kooperationen mit dem Trial Team des Centrums für Schlaganfallforschung (Leitung: Prof. Dr. Christian Nolte), der Kliniken für Neurologie der Charité, sowie der Asklepios Klinikums Brandenburg (Amendement aus August 2021: Geändert zu Klinik Hennigsdorf aufgrund des Ortwechsels des kooperierenden Arztes) und den Stroke Units (und Intensivstation: Amendement aus August 2021) der Charité genutzt. Nach initialem Screening der teilnahmeberechtigten Patient*innen, werden Patient*innen vom Studienpersonal aktiv angesprochen. Neben der mündlichen Aufklärung erhalten die Patient*innen und/oder Angehörigen zudem eine schriftliche Studieninformation.<br>Zudem wird durch Flyer und ggf. Aushängen in den Stationen auf die Studie aufmerksam gemacht. Hier wird die Studie in zielgruppengerechter Weise vorgestellt und auch die Möglichkeit der Kontaktaufnahme durch den/die Patient*in und/oder Angehörigen ermöglicht durch Angabe von Kontaktinformationen zur Studienkoordination.<br>Im Rahmen dieses Vorgehens können Teilnehmende in den RCT und die Kohortenstudie eingeschlossen werden. (Amendement aus Januar 2022: aufgrund der erschwerten Pandemiesituation mit Zugangsbeschränkungen, wurde die Rekrutierung durch verstärktes Verteilen von Informationsmaterial durchgeführt. Patient:innen, die nicht erreicht werden konnten, wurden postalisch kontaktiert mit einer Information zur Studie und Rückumschlag in Kooperation mit dem Trial Team des Centrums für Schlaganfallforschung.<br>Außerdem wurde der Studieneinschluss von Patient:innen und Angehörigen ermöglicht, welche aktiv das Studienteam kontaktieren – unabhängig vom Rekrutierungszentrum)<br><br>Lungenkrebs:<br>Zur Kontaktherstellung und Rekrutierung der Studienteilnehmenden wird die bereits bestehende Kooperation mit dem Charité Comprehensive Cancer Center, der Lungenkrebs-Ambulanz der Charité und dem Städtischen Klinikum Brandenburg genutzt.

	Nach initialem Screening der teilnahmeberechtigten Patient*innen, werden die Patient*innen durch das Studienpersonal in der Ambulanz oder Station aktiv angesprochen. Neben der mündlichen Aufklärung erhalten die Patient*innen und/oder Angehörigen zudem eine schriftliche Studieninformation.<br>Zudem wird durch Flyer und ggf. Aushänge und Aufsteller an den Rekrutierungsorten auf die Studie aufmerksam gemacht. Hier wird die Studie in zielgruppengerechter Weise vorgestellt und auch die Möglichkeit der Kontaktaufnahme durch die Patient*innen und/oder Angehörigen ermöglicht durch Angabe von Kontaktinformationen zur Studienkoordination.<br>Im Rahmen dieses Vorgehens können Teilnehmende in den RCT und die Kohortenstudie eingeschlossen werden.
16. Ggf.: **Grund für die Einbeziehung und Darlegung des therapeutischen Nutzens für Personen, die minderjährig und/oder nicht einwilligungsfähig sind**.	Es werden keine minderjährigen Personen in die Studie eingeschlossen.
17. Beziehung zwischen Studienteilnehmer und Studienarzt/-ärztin (Ist der Studienarzt zugleich der behandelnde Arzt?)	Entfällt.
18. Erklärung zur Einbeziehung möglicherweise vom Sponsor abhängiger Personen	Trifft nicht zu. Es werden keine vom Sponsor abhängigen Personen in die Studie eingeschlossen.
19. Maßnahmen, die eine Feststellung zulassen, ob ein Studienteilnehmer an mehreren Studien zugleich oder vor Ablauf einer in der vorangegangenen Studie festgelegten Frist teilnimmt.<br>Ist die Teilnahme an mehreren Studien möglich?	Die Möglichkeit der Teilnahme an mehreren Studien ist prinzipiell möglich.<br>Ausgenommen ist die gleichzeitige Teilnahme an weiteren Studien:<br>• Wenn es sich um Interventionsstudien zur Evaluation neuer Versorgungsformen handelt bzw. die Studie den standardisierten Versorgungsablauf stark verändert<br><br>Durch Absprache mit den koordinierenden Einrichtungen für Studienteilnahmen der Studienzentren wird dies nach Einwilligung bei den Patient*innen abgefragt. Im Zweifel erfolgt eine Einzelfallprüfung, die Art und Umfang einer weiteren Studienteilnahme berücksichtigt.
20. Ggf.: Honorierung bzw. Kostenerstattung der Studienteilnehmer (Höhe, wofür soll gezahlt werden?)	Teilnehmer RCT und Kohorte:<br>Keine Honorierung geplant, außer Teilnehmende führen zu den üblichen Assessments der Studie noch ein qualitatives Interview (Dauer ca. 1 Stunde) durch. Dann ist die Zahlung einer Aufwandsentschädigung von 25 Euro geplant.
21. Ggf.: Plan für die Weiterbehandlung und medizinische Betreuung der betroffenen Personen nach dem Ende der Studie	Nach Abschluss der Studie nach 1 Jahr ist keine weitere Betreuung durch den/die Patientennavigator*in geplant und Patient*innen erhalten die üblichen Maßnahmen der Regelversorgung.<br>Teilnehmende erhalten zudem die NAVICARE Broschüre mit Unterstützungsangeboten.

22. Ggf.: Versicherung der Studienteilnehmer (Versicherungsbestätigung und Versicherungsbedingungen, Versicherer, Versicherungsumfang, Versicherungsdauer)	Durch allg. Betriebshaftpflichtversicherung der Charité und Medizinischen Hochschule Brandenburg abgedeckt.
23. Dokumentationsverfahren:<br>- Ggf. Verweis auf CRF-Bögen<br>-Angabe der zu erfassenden Daten<br>- Probenumgang<br>- Aufbewahrung / Archivierung (inkl. Fristen)<br>- Zugang zu den Daten und Proben	Eine detaillierte Tabelle mit den zu untersuchenden Indikatoren und den sich daraus ergebenden zu erhebenden Daten ist in Tabellen 1-3 dargestellt.<br><br>Für die Evaluation des Navigationsprozesses erhobene Daten sind in Abbildung 3 als Entwurf einer möglichen Erhebungsstruktur dargestellt. Diese Daten werden durch den/die Navigator*in im Rahmen ihrer allgemeinen Dokumentation schriftlich erhoben. Die Daten zur Evaluation werden durch den/die Navigator*in elektronisch übertragen.<br><br>RCT und Kohorte<br>Medizinische Daten des CRF werden durch das Studienpersonal aus den medizinischen Informationssystemen der Studienzentren extrahiert. Daten zu Hospitalisierungen im Verlauf der Studienteilnahme werden durch Abfrage der Entlassbriefe bei den Krankenhäusern erfragt.<br>Die Fragebogendaten werden durch schriftlich oder, bei Bedarf, durch telefonische Befragungen erhoben. Alle Daten werden elektronisch über das REDCap System gesammelt.<br>Die elektronisch gesammelten Daten werden über eine Netzwerkverbindung auf den REDCap-Server des Berliner Instituts für Gesundheitsforschung (BIG) übertragen. Die temporär auf dem mobilen Endgerät gespeicherten Daten werden durch die Anwendung verschlüsselt. In regelmäßigen Abständen erfolgt eine Löschung aller lokalen Daten der Anwendung. (siehe Betriebshandbuch REDCap in der Version 1.1 vom 17. Dezember 2015, Herausgabe nur durch Charité IT-Sicherheitsbeauftragte). Für das REDCap System liegt ein positives Datenschutzvotum von der Charité sowie Berliner Datenschutz vom 13.Jan. 2016 vor. Weltweit wird das open-source REDCap System in mehr als 1792 Einrichtungen und 240000 Studien/Projekten benutzt (www.project-redcap.org).<br><br>Zugriff zu identifizierenden Daten haben nur Mitglieder der Studienkoordination am Institut für Public Health. Daten werden an weitere Studienmitarbeiter*innen nur in pseudonymisierter Weise übertragen und ausgewertet.<br><br>Prozessevaluation:<br>**Interviews mit Studienteilnehmer*innen:**<br>Die Interviews mit Studienteilnehmer*innen werden mit einem Diktiergerät digital aufgezeichnet. Die Tonaufnahmen werden auf gesondert geschützten Bereichen des Servers der Medizinischen Hochschule Brandenburg gespeichert, pseudonymisiert transkribiert, mit der Analysesoftware MAXQDA® ausgewertet und nach Studienende mindestens 10 Jahre aufbewahrt. Die Publikation erfolgt mit

	pseudonymisierten Daten. Auf die Daten haben nur autorisierte Mitarbeiter*innen des Projekts Zugriff.<br><br>**Interviews mit Study Nurses und Navigator*innen:**<br>Die Interviews mit den Study Nurses und Navigator*innen des Projekts werden mit einem Diktiergerät digital aufgezeichnet. Die Tonaufnahmen werden auf gesondert geschützten Bereichen des Servers der Medizinischen Hochschule Brandenburg gespeichert, pseudonymisiert transkribiert, mit der Analysesoftware MAXQDA® verwaltet und nach Studienende mindestens 10 Jahre aufbewahrt. Die Publikation erfolgt mit pseudonymisierten Daten. Auf die Daten haben nur autorisierte Mitarbeiter*innen des Projekts Zugriff.<br><br>**Teilnehmende Beobachtung:**<br>Im Rahmen der teilnehmenden Beobachtung werden von der/dem wissenschaftlichen Mitarbeiter*in handschriftliche Protokolle angefertigt. Diese Protokolle werden in abgeschlossenen Schränken am Institut für Sozialmedizin und Epidemiologie der Medizinischen Hochschule Brandenburg aufbewahrt und sind nur autorisiertem Personal des Projekts zugänglich. Die handschriftlichen Protokolle werden anschließend in digitale Dokumente umgewandelt. Die digitalen Dokumente werden auf gesondert geschützten Servern der Medizinischen Hochschule Brandenburg gespeichert. Auf die Daten haben nur autorisierte Mitarbeiter*innen des Projekts Zugriff. Sowohl die handschriftlichen Protokolle als auch die digitalen Dokumente werden nach Studienende für mindestens 10 Jahre aufbewahrt.<br><br>**Routinedaten:**<br>Zur Sicherung der Daten werden – analog zur ersten Förderphase von CoreNAVI folgende Maßnahmen durchgeführt:<br>Die von der AOK Nordost übermittelten pseudonymisierten Versichertendaten werden auf einem Server der Charité verschlüsselt gespeichert. Der Datenzugang ist passwortgeschützt und nur ausgewählte Mitarbeiter*innen aus dem Projektteam des Institutes für Medizinische Soziologie und Rehabilitationswissenschaft möglich. Entsprechend guter Praxis Sekundärdatenanalyse (GPS) (Swart et al. 2015) werden die pseudonymisierten Daten über einen Zeitraum von 10 Jahren verschlüsselt gespeichert. Dies dient einer Rekonstruktion und gegebenenfalls Reproduktion der Analyseergebnisse zu einem späteren Zeitpunkt.<br>Zugriff zu identifizierenden Daten haben nur Mitglieder der Studienkoordination am Institut für Public Health.
24. Ggf.: Beschreibung, wie der Gesundheitszustand gesunder betroffener Personen dokumentiert werden soll	Entfällt

25. Ggf.: Methoden, unerwünschte Ereignisse festzustellen, zu dokumentieren und mitzuteilen (wann, von wem und wie ?)	Entfällt
26. Vorgehen zum Schutz der Geheimhaltung der gespeicherten Daten, Dokumente und ggf. Proben, Darlegung der Pseudonymisierung oder Anonymisierung der Daten und Proben von Studienteilnehmern **(Initialen und Geburtsdatum als Codierungsschema sind nicht zulässig!)**<br>- Beschreibung der Trennung von Krankenakten, Studiendokumentation und Zuordnung der personenbezogenen Daten<br><br>- Nennung der Zugriffsrechte einschließlich des Zugangs zu Teilnehmeridentifikations-listen während und nach der Studiendurch-führung<br>- Detaillierte Angabe der Verfahren für die Übertragung, Verschlüsselung, Einschränkung der Verarbeitung (Sperrung) und Löschung (einschließlich Angabe der ggf. verwendeten Netzstruktur und verwendete Server).<br>-ggf. Zugang zu identifizierenden Daten für gesetzlich berechtigte Prüfer (Dritte) zur zweckgebundenen Einsichtnahme in die dafür erforderlichen Akten.	Alle Studienteilnehmenden erhalten nach Studieneinschluss (ggf. Randomisierung) eine pseudonymisierte Teilnehmer-ID, welche keine identifizierenden Eigenschaften enthält. Die Erstellung der Teilnehmer-ID wird in einem automatisierten Prozess durchgeführt.<br><br>Alle Daten werden im Dokumentationssystem REDCap pseudonymisiert erfasst.<br><br>Originalstudiendokumente (Einwilligungen, Fragebögen) werden in abschließbaren Schränken für 10 Jahre gelagert. Hierbei werden identifizierbare Dokumente räumlich getrennt aufbewahrt.<br>Zugang zu identifizierenden Daten hat nur der/die Studienleiter*in sowie durch den/die Studienleiter*in direkt autorisierte Personen innerhalb der Studienkoordination des Institutes für Public Health.<br><br>Zur Erlangung der GKV-Routinedaten der Studienteilnehmenden des RCT wird der AOK eine verschlüsselte Liste mit relevanten identifizierenden Variablen übermittelt (Versichertennummer, Geburtsdatum, Studienpseudonym). Die AOK kann auf diesem Wege eine Identifizierung der Versicherten im GKV-Routinedatensatz vornehmen und die Daten der entsprechenden Versicherten extrahieren. Die Pseudonymisierung der Patientendaten der AOK wird durch den Dateneigner (AOK Nordost) unter Einhaltung datenschutzrechtlicher Vorschriften sichergestellt, indem das im Routinedatensatz vorhandene Identifizierungsmerkmal /Versichertennummer gelöscht, und durch das der AOK bekannte Studienpseudonym ersetzt wird. Zusätzlich erfolgt eine weitere Löschung von im Datensatz enthaltenen personenidentifizierenden Merkmalen (z.B. Name, Wohnort). Der nun vorliegende Datensatz enthält neben den Auswertungsvariablen nun nur noch das Studienpseudonym. Diese Versichertendaten werden von der AOK Nordost verschlüsselt und der Charité über einen Data Sharing Point zur Verfügung gestellt. Hier werden die Daten auf einem Passwort geschützten Server der Charité abgelegt. Nur ausgewählten Projektmitarbeiter*innen ist der Datenzugriff möglich. Da die Daten zu diesem Zeitpunkt noch über das Studienpseudonym verfügen, kann ein Linkage mit den in der Studie erhobenen Primärdaten (z.B. Lebensqualität) erfolgen. Im Anschluss erfolgt die Löschung des Studienpseudonyms, so dass die verknüpften Daten ab diesem Zeitpunkt als faktisch anonym angesehen werden können. In dieser Form werden verknüpfte Datensatze den auswertenden Einrichtungen zur Verfügung gestellt.

27. Erklärung zur Einhaltung des Datenschutzes<br>- Zusicherung, dass alle über den Studien-teilnehmer erhobenen und gespeicherten Daten vertraulich (Datengeheimnis und ärztliche Schweigepflicht) behandelt werden.<br>- Zusicherung, dass die identifizierenden Daten nur dem Studienleiter oder von ihm beauftragten Mitarbeitern zugänglich sind.<br>- Angabe der Maßnahmen zur Sicherstellung der Vertraulichkeit<br>- Maßnahmen zur datenschutzgerechten Übermittlung von Daten, die für Dritte keinen Personenbezug herstellen lassen.<br>- Angaben zu Auskunfts-, Widerrufs-, Berichtigungs- und Löschmöglichkeiten,<br>- Maßnahmen zur Sicherstellung der Rechte der Teilnehmer.<br>- Falls Übermittlungen ins Nicht-EU-Ausland vorgesehen sind: Maßnahmen zur Einhaltung des Datenschutzes (z.B. Vorliegen eines Angemessenheitsbeschlusses der EU-Kommission oder explizite Einwilligung der Studienteilnehmer in solche Übermittlungen	Der Umgang mit den erhobenen Daten unterliegt den Datenschutzgesetzen des zuständigen Bundeslandes Berlin (BlnDSG), Brandenburg (BbgDSG) und dem Datenschutz durch das SGB X sowie der Datenschutzgrundverordnung (DSGVO). Nach Standard der Guten Wissenschaftlichen Praxis erfolgt die Speicherung über einen Zeitraum von 10 Jahren. Die Daten werden vertraulich behandelt und eine Übermittlung der Daten an unbefugte Dritte ist ausgeschlossen. Zur Sicherstellung der Vertraulichkeit werden alle erhobenen Daten nur auf den in Deutschland stehenden Servern der Charité und der Medizinischen Hochschulde Brandenburg (für qualitative Daten) gespeichert. Eine Übermittlung von Daten ins Ausland findet nicht statt. Die Daten sind nur dem/der Studienleiter*in und dem von ihm/ihr beauftragten Studienpersonal zugänglich.<br>Zugang zu identifizierenden Daten hat nur die Studienleitung sowie durch die Studienleitung autorisierte Personen der Studienkoordination am Institut für Public Health. Hierdurch werden die Auskunfts-, Widerrufs-, Berichtigungs- und Löschrechte ermöglicht.<br>Die Daten können von den Studienmitarbeiter*innen außerhalb der Studienkoordination am Institut für Public Health nicht auf einzelne Personen zurückverfolgt werden.<br><br>Das Datenschutzkonzept wird parallel zur Stellung des Antrages mit den Datenschutzbeauftragten der Charité und der AOK Nordost abgestimmt.
28. Namen und Anschriften der Einrichtungen, die als Studienzentrum oder Studienlabor in die Studie eingebunden sind, sowie der Studienleiter und der Studienärzte<br>- Angabe beteiligter externer Dienstleister mit Angabe der Datenzugriffsmöglichkeit	Priv.-Doz. Dr. Nina Rieckmann (Studienleitung), Institute of Public Health, Charité-Universitätsmedizin Berlin, Berlin (bis Juli 2022)<br><br>Prof. Dr. Christine Holmberg (Studienleitung) Medizinische Hochschule Brandenburg Theodor Fontane, Brandenburg/Havel<br><br>Priv.-Doz. Dr. Dipl.-Psych. Ute Goerling, Charité Comprehensive Cancer Center, Charité-Universitätsmedizin Berlin, Berlin<br><br>Prof. Dr. Andreas Meisel, Center for Stroke Research Berlin, Department of Neurology, Charité-Universitätsmedizin Berlin, Berlin<br><br>Priv.-Doz. Dr. Susanne Schnitzer, Institute of Medical Sociology and Rehabilitation Science, Charité-Universitätsmedizin Berlin, Berlin<br><br>Priv.-Doz. Dr. Ulrike Grittner, Institut für Biometrie und Klinische Epidemiologie, Charité – Universitätsmedizin Berlin, Berlin<br><br>Prof. Dr. Thomas Reinhold<br>Institut für Sozialmedizin, Epidemiologie und Gesundheitsökonomie<br>Charité – Universitätsmedizin Berlin, Berlin

	<u>Rekrutierende Studienzentren:</u><br><br>Schlaganfall:<br>Klinik für Neurologie Charité/Centrum für Schlaganfallforschung<br>Charité Universitätsmedizin Berlin<br>Prof. Dr. Andreas Meisel<br><br>Asklepios Fachklinikum Brandenburg<br>Klinik für Neurologie<br>(Amendement aus August 2021: Geändert zu Klinik Hennigsdorf aufgrund des Ortwechsels des kooperierenden Arztes)<br>Prof. Dr. Stephan Schreiber<br><br>Lungenkrebs:<br>Lungentumorambulanz<br>Charité Lungenkrebszentrum<br>Charité Universitätsmedizin Berlin<br>Dr. med. Nikolaj Frost<br><br>Städtisches Klinikum Brandenburg<br>Zentrum für Innere Medizin II<br>Prof. Dr. med. P. Markus Deckert<br><br><u>Wissenschaftliche Mitarbeiter*innen:</u><br>Hella Fügemann<br>Dr. Kathrin Gödde<br>Raphael Kohl
29. Angaben zur Eignung der Prüfstelle, insbesondere zur Angemessenheit der dort vorhandenen Mittel und Einrichtungen sowie des zur Durchführung der klinischen Prüfung zur Verfügung stehenden Personals und zu Erfahrungen in der Durchführung ähnlicher Studien	Das multiprofessionelle Wissenschaftler-Team (Ärzt*innen, Epidemiolog*innen, Anthropolog*innen, Psycholog*innen, Gesundheitswissenschaftler*innen) am Institut für Public Health verfügt über langjährige und umfassende Expertise und Kompetenz in der Planung, Durchführung und Auswertung von epidemiologischen und qualitativen Studien. Forschungsschwerpunkte sind Herz-Kreislauf- sowie Krebserkrankungen im Zusammenhang mit Prozessen der Entscheidungsfindung, gesundheitsbezogener Lebensqualität und patienten-zentrierter Versorgungsforschung.<br>Auch die an der Studie beteiligten Institutionen verfügen über umfangreiche und langjährige Erfahrungen in der Planung und Umsetzung von Forschungsprojekten.
30. Vereinbarung über den Zugang des Prüfers/Hauptprüfers/Leiters der klinischen Prüfung, zu den Daten und den Grundsätzen über die Publikation.<br>- Publikationen in einer Form, die keinen Rückschluss auf die Person zulässt.	Die Studienleiterin hat vollen Zugang zu den erhobenen Daten. Die Publikation der Studienergebnisse in nationalen und internationalen Zeitschriften mit Peer-Review-Verfahren sowie in Vorträgen auf Kongressen ist geplant. Es können bei der Präsentation der Ergebnisse keine Rückschlüsse auf Einzelpersonen gezogen werden.
31. Angaben zur Finanzierung der Studie: Finanzierungsquelle (Name und Sitz) und Höhe der Förderung in €.<br>-ggf. Angabe der Kostenstelle zur ILV Abrechnung der Gebühr	Bundesministerium für Bildung und Forschung<br>[Adresse and und Höhe der Förderung für PLOS One Publikation gelöscht]
